# The GAR domain integrates functions that are necessary for the proper localization of fibrillarin (FBL) inside eukaryotic cells

**DOI:** 10.7717/peerj.9029

**Published:** 2020-04-28

**Authors:** Maria Y. Shubina, Eugene A. Arifulin, Dmitry V. Sorokin, Mariya A. Sosina, Maria A. Tikhomirova, Marina V. Serebryakova, Tatiana Smirnova, Svyatoslav S. Sokolov, Yana R. Musinova, Eugene V. Sheval

**Affiliations:** 1 Belozersky Institute of Physico-Chemical Biology, Lomonosov Moscow State University, Moscow, Russia; 2 Laboratory of Mathematical Methods of Image Processing, Faculty of Computational Mathematics and Cybernetics, Lomonosov Moscow State University, Moscow, Russia; 3 Koltzov Institute of Developmental Biology, Russian Academy of Sciences, Moscow, Russia; 4 Faculty of Bioengineering and Bioinformatics, Lomonosov Moscow State University, Moscow, Russia; 5 Department of Cell Biology and Histology, Faculty of Biology, Lomonosov Moscow State University, Moscow, Russia; 6 Skobelkin State Scientific Center of Laser Medicine FMBA, Moscow, Russia; 7 LIA 1066 LFR2O French-Russian Joint Cancer Research Laboratory, Villejuif, France

**Keywords:** Nucleus, Nucleolus, Fibrillarin (FBL), GAR domain, NLS, NoLS, Methylation

## Abstract

Fibrillarin (FBL) is an essential nucleolar protein that participates in pre-rRNA methylation and processing. The methyltransferase domain of FBL is an example of an extremely well-conserved protein domain in which the amino acid sequence was not substantially modified during the evolution from *Archaea* to *Eukaryota*. An additional N-terminal glycine–arginine-rich (GAR) domain is present in the FBL of eukaryotes. Here, we demonstrate that the GAR domain is involved in FBL functioning and integrates the functions of the nuclear localization signal and the nucleolar localization signal (NoLS). The methylation of the arginine residues in the GAR domain is necessary for nuclear import but decreases the efficiency of nucleolar retention via the NoLS. The presented data indicate that the GAR domain can be considered an evolutionary innovation that integrates several functional activities and thereby adapts FBL to the highly compartmentalized content of the eukaryotic cell.

## Introduction

Eukaryotic cells contain numerous organelles with or without a membrane(s) that are effectively compartmentalized the cellular processes. Nuclear proteins are translated in the cytoplasm and, therefore, sophisticated mechanisms of nuclear import have evolved. Proteins larger than ~40–60 kDa are selectively transferred by an energy-dependent mechanism that requires additional transport factors, called karyopherins, which recognize the nuclear localization signals (NLSs) of their cargo proteins. Inside nuclei, proteins can accumulate inside membrane-free organelles, which are usually referred to as nuclear bodies. Some nuclear bodies have complex organization. For example, the nucleolus is composed of three subcompartments, namely, the fibrillar center (FC), the dense fibrillar component (DFC), and the granular component (GC). The nucleolus demonstrates vectoral organization: transcription of pre-rRNA occurs at the border of the FCs and DFC, pre-rRNA processing and pre-ribosome biogenesis occur in the DFC and GC. The origin and evolution of the mechanisms that lead to effective compartmentalization of nuclear proteins (i.e., the effective nuclear import and subsequent accumulation inside nuclear bodies) are not obvious. Here, we investigate the possible mechanisms that enabled proteins to adapt to the highly compartmentalized content of eukaryotic cells, focusing on essential nucleolar protein fibrillarin (FBL) as an example.

FBL localizes preferentially in the DFC of the nucleolus, where active ribosomal DNA transcription and the early stages of rRNA processing take place (De Carcer & Medina, 1999). FBL catalyzes the 2′-O-methylation of pre-rRNA, participates in pre-rRNA processing (Tollervey et al., 1993), and regulates rRNA transcription by methylation of histone H2A (Tessarz et al., 2014; Loza-Muller et al., 2015). Accumulated experimental data suggest that FBL, one of the key components of ribosome formation, can influence various cellular processes, as well as the development of pathological processes and even the dynamics of aging (see reviews: Monaco et al., 2018; Shubina, Musinova & Sheval, 2018).

FBL is a relatively small protein (34–38 kDa) that consists of three domains—an N-terminal glycine–arginine-rich (GAR) domain (80 a.a.), an RNA-binding domain (~90 a.a.) and a C-terminal α-helical domain (~33 a.a.) (Aris & Blobel, 1991). The RNA-binding domain and the α-helical domain constitute an AdoMet-dependent methyltransferase (MT) domain. The amino acid sequence, three-dimensional structure and function of the MT domain have been remarkably conserved throughout the evolution from *Archaea* to human (for reviews see: Rodriguez-Corona et al., 2015; Shubina, Musinova & Sheval, 2016). The most reliable data indicating the conservation of FBL function were presented in experiments based on the complementation of yeast FBL (NOP1) knock-down by other eukaryotic FBLs: human (Jansen et al., 1991), *Xenopus* (Jansen et al., 1991), *Arabidopsis* (Barneche, Steinmetz & Echeverría, 2000) (but not *Tetrahymena* (David et al., 1997)). FBLs could functionally replace NOP1 because of the outstanding functional conservativity of this protein.

The functional role of the GAR domain is unclear. Experiments with temperature-sensitive lethal point mutations in yeast FBL (encoded by the *nop1* gene) indicated that all major posttranscriptional activities in ribosome synthesis, such as pre-rRNA processing, pre-rRNA modification and ribosome assembly, were dependent on NOP1 (Tollervey et al., 1993), but to our knowledge, the role of the GAR domain in these FBL functions has not been investigated. FBL can modulate the activity of other proteins interacting with them via the GAR domain. For example, it was demonstrated that sirtuin 7—a protein that is essential for 45S pre-rRNA cleavage—physically interacts with Nop56 and the GAR domain of FBL (Sirri et al., 2019). Recently, novel ribonuclease activity confined to the GAR domain was described (Rodriguez-Corona et al., 2017). This activity is likely necessary for some stage(s) of pre-rRNA processing.

Nucleoli are formed via liquid–liquid phase separation (Correll, Bartek & Dundr, 2019) and have several distinct phase-separated layers (Feric et al., 2016; for review see: Sawyer, Sturgill & Dundr, 2019). Recent data also indicate that the interaction between FBL and nascent pre-rRNA promotes DFC assembly via a liquid–liquid phase separation mechanism (Yao et al., 2019). Local self-association of FBL molecules via the GAR domain leads to phase-separated clusters in which the pre-rRNA molecules are temporarily immobilized, thus facilitating pre-rRNA processing and DFC formation.

It seems that the GAR domain is also involved in targeting FBL to nuclei and nucleoli. It was demonstrated that the GAR domain was necessary for targeting FBL to nucleoli (Pih et al., 2000; Snaar et al., 2000; Levitskiy et al., 2004; Zheng et al., 2016). For example, Snaar and coauthors demonstrated that the GAR domain contained a nucleolar localization signal (NoLS) that was not required to target FBL to the DFC but appeared to increase the efficiency of FBL targeting to nucleoli (Snaar et al., 2000).

The GAR domain in FBL contains several RGG motifs alternated with a limited number of phenylalanine residues (Aris & Blobel, 1991). Arginine residues within the GAR domain seem to undergo posttranslational asymmetrical dimethylation (Lischwe et al., 1985; Najbauers et al., 1993; Liu & Dreyfuss, 1995; Ai et al., 1999; Yagoub et al., 2015). For example, human FBL is a heavily methylated nucleolar protein that contains 4.1% N^G^,N^G^-dimethylarginine (Lischwe et al., 1985). It has been demonstrated that the N-terminal fragment of the FBL molecule is a substrate for PRMT1 (Tang et al., 1998) and PRMT7 (Zurita-Lopez et al., 2012). It is not clear if there is any functional significance for the arginine methylation of the GAR domain in FBL. In general, asymmetric arginine methylation contributes to signal transduction, nuclear localization and interaction with nucleic acids (Gary & Clarke, 1998), and methylation of arginine residues in eukaryotic proteins may regulate their interaction with RNA (Tao & Frankel, 1992; Liu & Dreyfuss, 1995; Gary & Clarke, 1998; McBride & Silver, 2001).

Additionally, it was also demonstrated that the hordeiviral movement protein encoded by the first gene of the triple gene block (TGBp1) of *Poa semilatent virus* (PSLV) (Semashko et al., 2012), *barley stripe mosaic virus* (BSMV) Triple Gene Block 1 (TGB1) protein (Li et al., 2018) and p2 of *Rice stripe virus* (Zheng et al., 2018) interact with GAR-domain of FBL of *Nicotiana benthamiana*, and this interactions play a direct role in cell-to-cell movement (Li et al., 2018).

The GAR domain is missing in *Archaea* homologs of eukaryotic FBL (FBL-like proteins) (Amiri, 1994), indicating that the GAR domain may be implicated in some functions that evolved after the origin of the cell nucleus as an adaptation of this extremely conserved archaeal protein to functioning within the eukaryotic cell. Here, we investigate the possible roles of the GAR domain and the methylated arginine residues within the GAR domain in FBL function. We used yeast complementation assay which was widely used for demonstration of functional activity of FBL and experiments with HeLa cells to investigate FBL localization in human cells. Our data indicate that GAR domain is necessary for FBL functioning and additionally, integrates NLS and NoLS. The nuclear accumulation of FBL depends on the methylation of arginine; in contrast, the nucleolar FBL accumulation decreases upon arginine methylation.

## Materials and Methods

### Plasmids

The following plasmids were used for the plasmid construction and site-directed mutagenesis: TagRFP-FBL (Svistunova et al., 2012), EGFP-NPM1 (Addgene plasmid 17578) (Wang et al., 2005), NPM1-TagRFP (Musinova et al., 2015), pYep368 (Myers et al., 1986), cDNA encoding *fbl* gene (Invitrogen) and EGFP-N1 (Clontech, Mountain View, CA, USA).

For FBL-EGFP, GAR-EGFP and ΔGAR-EGFP plasmid construction, the region encoding human FBL and its domains (GAR and ΔGAR) was amplified from cDNA using the primers presented in Table S1. The resulting PCR product was digested by BamHI and HindIII and cloned into the pEGFP-N1 vector (Clontech, Mountain View, CA, USA).

For pFBL-LacZ-EGFP plasmid construction, the region encoding human FBL was amplified from cDNA (Table S1). The PCR product was digested by BglII and SalI, gel purified and cloned into the pEGFP-N1 vector (Clontech, Mountain View, CA, USA). LacZ was amplified from the pYep368 vector (Table S1). The resulting PCR product was digested by KpnI and BamHI, purified and cloned into the previously obtained pFBL-EGFP plasmid.

To generate the pLacZ-EGFP plasmid, the region encoding β-galactosidase was amplified by PCR from the pCMV-LacZ plasmid (Table S1). The resulting PCR product was digested by HindIII and BamHI, gel purified and cloned into the pEGFP-N1 vector (Clontech, Mountain View, CA, USA).

To obtain the NLS^SV40^-EGFP plasmid, we designed adapters encoding the NLS from the T antigen of SV40 virus with the necessary restriction sites (Table S1). The oligonucleotides were incubated in annealing buffer (10 mM Tris, pH 8.0; 50 mM NaCl; and 1 mM EDTA) for 5 min at 94 °С and then for 5 min at 70 °С. The resulting product was BamHI and SalI digested and cloned into the pEGFP-N1 vector (Clontech, Mountain View, CA, USA).

Point mutations of the FBL DNA sequence were made using the Change-IT Multiple Mutation Site-Directed Mutagenesis Kit (Affymetrix, Santa Clara, CA, USA) according to the manufacturer’s instructions. Some amino acid residues were combined into loci to facilitate mutation (Fig. S1). Mutations were introduced sequentially; each subsequent round of mutagenesis was carried out after confirming the presence of the previously necessary mutation by sequencing.

### Yeast complementation assay

To place the *nop1* gene under the control of a regulatable galactose promotor GAL1, we designed primers in which the 5′-ends (~40 nucleotides) were complementary to the desired target gene sequence (NOP1) and the 3′-ends (~20 nucleotides) were annealed to the selected marker gene (His3MX6) and regulatable promotor (pGAL). We used plasmid pFA6a-His3MX6-pGAL1 (Longtine et al., 1998) as a template for PCR. The amplified linear DNA was purified, and the wild-type W303 yeast strain was transformed using 0.1 M LiAc buffer (Gietz, 2014). The transformed cells were spread on selective medium YNB RafGal-His. His+ transformants were picked and streaked on YNB-His (D) plates. This strain was referred to as GAL-NOP1. Yeast genomic DNA was isolated from cells and subsequent PCR was performed as a control to check for the presence of the desired construct (pGAL-NOP1).

For the amplification of human FBL, primers were synthesized for all the domains (GAR and ΔGAR) and mutant forms of FBL, in which all arginine residues in the GAR domain were replaced by alanine residues (R⇒A) or lysine residues (R⇒K). Human FBL cDNA and the mutated forms of FBL were used as templates. The amplified products were purified, digested by BamHI and HindIII and ligated into the p416GPD yeast vector (Musti et al., 1983; Bitter & Egan, 1984; Mumberg, Mailer & Funk, 1995). The resulting plasmids were referred to as pGPD-FBL, pGPD-ΔGAR, pGPD-GAR, pGPD-GAA-FBL and pGPD-GAK-FBL.

The *nop1* gene was amplified with PCR using total yeast genomic DNA as a template. The amplified product was purified, digested by BamHI and HindIII and ligated into the p416GPD yeast vector. The resulting plasmid was referred to as pGPD-NOP1. GAL-NOP1 cells were transformed with pGPD-FBL, pGPD-ΔGAR, pGPD-GAR, pGPD-GAA-FBL, pGPD-GAK-FBL or pGPD-NOP1 plasmid; the transformed cells were spread onto the selective medium YNB-Ura, Gal. All newly generated strains were verified by PCR with independently designed primers (Table S1).

The obtained yeast strains (Table 1) were grown overnight at 23 °C on YNB-His (D) plates to deplete endogenous NOP1. The next day, the cells of each strain were resuspended in deionized water at a density of OD550 = 0.1 in a total volume of one ml (~1·10^6^ cells/ml). Suspensions of each strain were spotted on YNB-Ura (D) and YNB-Ura (Gal) with 10× dilutions and grown for 2–5 days at 23 °C. The number of colony forming units (CFUs) in each spot was counted and the obtained data were statistically analyzed. To set a baseline of 100%, we took the number of CFUs on the YNB-Ura (Gal) medium. Yeast survival was evaluated as the ratio of the CFUs on a medium with glucose to the CFUs on a medium with galactose.

**Table 1:** Strains used in this study.

Strain	Genotype	Source
W303-1A	*MATa ade2-101 his3-11 trp1-1 ura3-52 can1-100 leu2-3*	Laboratory of A. Hyman (Severin & Hyman, 2002)
GAL-NOP1	*MATa ade2-101 his3-11 trp1-1 ura3-52 can1-100 leu2-3 P*_*GAL*_*-NOP1::HIS3MX6*	This study
FBL	*MATa ade2-101 his3-11 trp1-1 ura3-52 can1-100 leu2-3 P*_*GAL*_*-NOP1::HIS3MX6 [URA3 P*_*GPD*_*-FBL]*	This study
GAR	*MATa ade2-101 his3-11 trp1-1 ura3-52 can1-100 leu2-3 P*_*GAL*_*-NOP1::HIS3MX6 [URA3 P*_*GPD*_*-GAR]*	This study
∆GAR	*MATa ade2-101 his3-11 trp1-1 ura3-52 can1-100 leu2-3 P*_*GAL*_*-NOP1::HIS3MX6 [URA3 P*_*GPD*_*-∆GAR]*	This study
GPD	*MATa ade2-101 his3-11 trp1-1 ura3-52 can1-100 leu2-3 P*_*GAL*_*-NOP1::HIS3MX6 [URA3]*	This study
NOP1	*MATa ade2-101 his3-11 trp1-1 ura3-52 can1-100 leu2-3 P*_*GAL*_*-NOP1::HIS3MX6 [URA3 P*_*GPD*_*-NOP1]*	This study
GAA-FBL	*MATa ade2-101 his3-11 trp1-1 ura3-52 can1-100 leu2-3 P*_*GAL*_*-NOP1::HIS3MX6 [URA3 P*_*GPD*_*-FBL(R→A)]*	This study
GAK-FBL	*MATa ade2-101 his3-11 trp1-1 ura3-52 can1-100 leu2-3 P*_*GAL*_*-NOP1::HIS3MX6 [URA3 P*_*GPD*_*-FBL(R→K)]*	This study
GAL-NOP1 *hmt1∆*	*MATa ade2-101 his3-11 trp1-1 ura3-52 can1-100 leu2-3 P*_*GAL*_*-NOP1::HIS3MX6 hmt1∆::kanMX*	This study
FBL *hmt1∆*	*MATa ade2-101 his3-11 trp1-1 ura3-52 can1-100 leu2-3 P*_*GAL*_*-NOP1::HIS3MX6 hmt1∆::kanMX [URA3 P*_*GPD*_*-FBL]*	This study
*hmt1∆*	*MATa ade2-101 his3-11 trp1-1 ura3-52 can1-100 leu2-3 hmt1∆::kanMX*	This study

Disruption of HMT1 in GAL-NOP1 yeast strain was performed as described by Gueldener with coathors (Gueldener et al., 2002). A PCR strategy was used to construct a DNA fragment in which KANMX is flanked by the 55 nucleotides before and 53 nucleotides after the target sequence of HMT1. The marker plasmid pUG6 served as a template for PCR to generate a gene disruption cassette using the primers presented in Table S1. Following PCR amplification, the reaction mixture was directly transformed into pGAL-NOP1 yeast strain using 0.1 M LiAc buffer (Gietz, 2014) to disrupt one copy the genomic *hmt1* by homologous recombination. The transformed cells were spread on selective medium YPGal + G418. Generated strains were verified by PCR. Verified yeast strain pGAL-NOP1 *hmt1*Δ was transformed with earlier obtained pGPD-FBL; the transformed cells were spread onto the selective medium YNB-Ura, Gal.

### RT-PCR

RNA was isolated from yeast cells using the hot formamide extraction method (Shedlovskiy, Shcherbik & Pestov, 2017). RNA quality and quantity were measured using the NanoPhotometer (Implen, Munich, Germany). Samples were treated with DNase I (Thermo Scientific, Waltham, MA, USA). First-strand cDNA synthesis was carried out with 1 µg of total RNA using the iScript cDNA Synthesis kit (BioRad, Hercules, CA, USA) according to the manufacturer’s instructions. PCR was performed in the T100 Thermal Cycler (BioRad, Hercules, CA, USA) using Phusion Hot Start II High-Fidelity PCR Master Mix (Thermo Scientific, Waltham, MA, USA) and the primers presented in Table S1.

Electrophoresis of PCR products was performed in 1.5% agarose gel. The gels were visualized in ChemiDoc Gel Imaging System (BioRad, Hercules, CA, USA).

### Cell culture

Human carcinoma cells (HeLa, The Russian collection of human, animals and plants cell cultures, Institute of Cytology, St. Petersburg, Russia) were grown in Dulbecco’s modified Eagle’s medium (Paneco, Moscow, Russia) supplemented with 10% heat-inactivated fetal bovine serum (HyClone, Logan, UT, USA), 2 mM L-glutamine (Paneco, Moscow, Russia) and antibiotic-antimycotic solution (Gibco, Waltham, MA, USA). To inhibit RNA transcription, the cells were grown in medium with 0.1 μg/ml actinomycin D (ActD). Cellular transfection was performed using the Lipofectamine 2000 reagent (Invitrogen, Waltham, MA, USA) according to the manufacturer’s instructions.

### ATP depletion assay

ATP depletion was carried out as described by Bhattacharya et al. (2006). Briefly, HeLa cells were grown in 35 mm dishes on a coverslip (MatTek, Ashland, MA, USA). The cells were treated with 20% Hank’s solution for 15 min and then transferred into Medium 1 (150 mM NaCl, 5 mM KCl, 1 mM CaCl_2_, 1 mM MgCl_2_ and 20 mM HEPES, pH 7.4) containing 10 mM sodium azide and 6 mM 2-deoxy-D-glucose and incubated for 50 min. Live cell imaging was performed with a Nikon C2 confocal microscope and a 60× Plan-Apo objective (NA 1.4) at 37 °C; focus stabilization was performed using the PFS system (Nikon, Minato City, Tokyo, Japan).

### Measurement of nuclear and nucleolar accumulation of the protein

To evaluate the nuclear and nucleolar accumulation of the protein, images of at least 50 living HeLa cells expressing EGFP-fused proteins were taken from two different experiments using a Nikon C2 confocal laser scanning microscope. Cells expressing different EGFP-fused proteins were imaged under identical conditions in all experiments. The regions of interest in the nucleolus, the nucleoplasm and cytoplasm were selected manually and the mean intensity of the fluorescence was measured using ImageJ software (http://rsbweb.nih.gov/ij/). The mean fluorescence intensity in the nucleolus (F_n_), nucleoplasm (F_nuc_) and cytoplasm (F_cyt_) were estimated to measure the concentration of each fusion protein. The ratios of the nucleoplasmic EGFP concentration to the cytoplasmic EGFP concentration (F_nuc_/F_cyt_) and the nucleolar EGFP concentration to the nucleoplasmic EGFP concentration (F_n_/F_nuc_) were used to measure the nuclear and nucleolar accumulation, respectively. Statistical analysis and preparation of the graphs were performed using Prism 6 software (GraphPad, San Diego, CA, USA).

### Fluorescence recovery after photobleaching

For the FRAP experiments, the cells were grown in 35 mm dishes containing coverslips. The medium was overlaid with mineral oil before the experiment, and the dishes were mounted into a Nikon A1 confocal microscope. Four individual scans were acquired for the FRAP experiments followed by a single pulse for photobleaching. The recovery curves were generated from images with the background subtracted. The relative fluorescence intensity (RFI) was calculated as RFI = *T*_0_*I_t_*/*T_t_I*_0_, where *T*_0_ is the total cellular intensity during prebleaching, *T_t_* is the total cellular intensity at time point *t*, *I*_0_ is the average intensity in the region of interest during prebleaching and *I_t_* is the average intensity in the region of interest at time point *t*. The results for 10–15 cells were averaged to obtain the final curve of fluorescence recovery.

### Inhibition of methylation

To inhibit methylation, the transfected cells were grown in medium with 2 μg/ml AdOx. The cells were analyzed the next day with a Nikon C1 confocal microscope.

### Lentivirus production and transduction

HEK 293 cells were cotransfected with the created lentivector construct coding FBL-FLAG and the lentiviral packaging plasmids using Lipofectamine 2000 reagent (Invitrogen, Carlsbad, CA, USA) according to the manufacturer’s instructions. 48 h after transfection, the supernatant containing the lentiviruses was collected, filtered through a 0.2 µm filter and immediately used to infect recipient HeLa cells. To increase the efficiency of the transduction, Polybrene (8 µg/ml) was added to the medium with pseudoviral particles. After infection, the cells were selected with puromycin (1 µg/ml).

### MALDI MS

FBL-FLAG was immunoprecipitated using FLAG immunoprecipitation kit (Sigma, Kawaski, Kanagawa, Japan). Pieces of about 2 mm^3^ of the Coomassie-stained gel were destained twice with 40% acetonitrile, 20 mM NH_4_HCO_3_ solution, dehydrated with 200 μl of 100% acetonitrile and rehydrated with 5 μl of the 20 mM NH_4_HCO_3_ solution (pH 7.5) containing 15 µg/ml enzyme. The proteins were digested either with trypsin for 6 h at 37 °C or with Lys-C for 4 h at 37 °C. The resulting peptides were extracted with 10 μl of 20% acetonitrile, 0.5% TFA solution and subjected to MALDI-MS analysis. Aliquots of 1 μl of the samples were mixed on a steel target with an 0.5 μl of 2,5-dihydroxybenzoic acid (Sigma-Aldrich, St. Louis, MO, USA) solution (30 mg/ml in 30% acetonitrile, 0.5% TFA). Spectra were recorded on an UltrafleXtreme MALDI-TOF/TOF mass spectrometer (Bruker Daltonik, Billerica, MA, USA) equipped with an Nd laser. For tryptic maps, the monoisotopic MH+ were measured in the reflection mode; the accuracy of mass peak measurement was within 30 ppm. For a number of tryptic fragments, MS/MS analysis was performed. Fragmentation spectra were obtained in the LIFT mode, the accuracy of daughter ion measurement was within 0.5 Da. Spectra of N-terminal peptides after Lys-C proteolysis were obtained in a linear mode; the accuracy of the measured average masses was within 1 Da. Peptide identification was carried out on the combined MS + MS/MS data using the Mascot search service (www.matrixsciene.com) in the SwissProt database. Candidate proteins having a confidence parameter of score >80 were considered certain reliably (*p* < 0.05).

## Results

### The GAR domain is involved in FBL functioning

**Figure 1:**
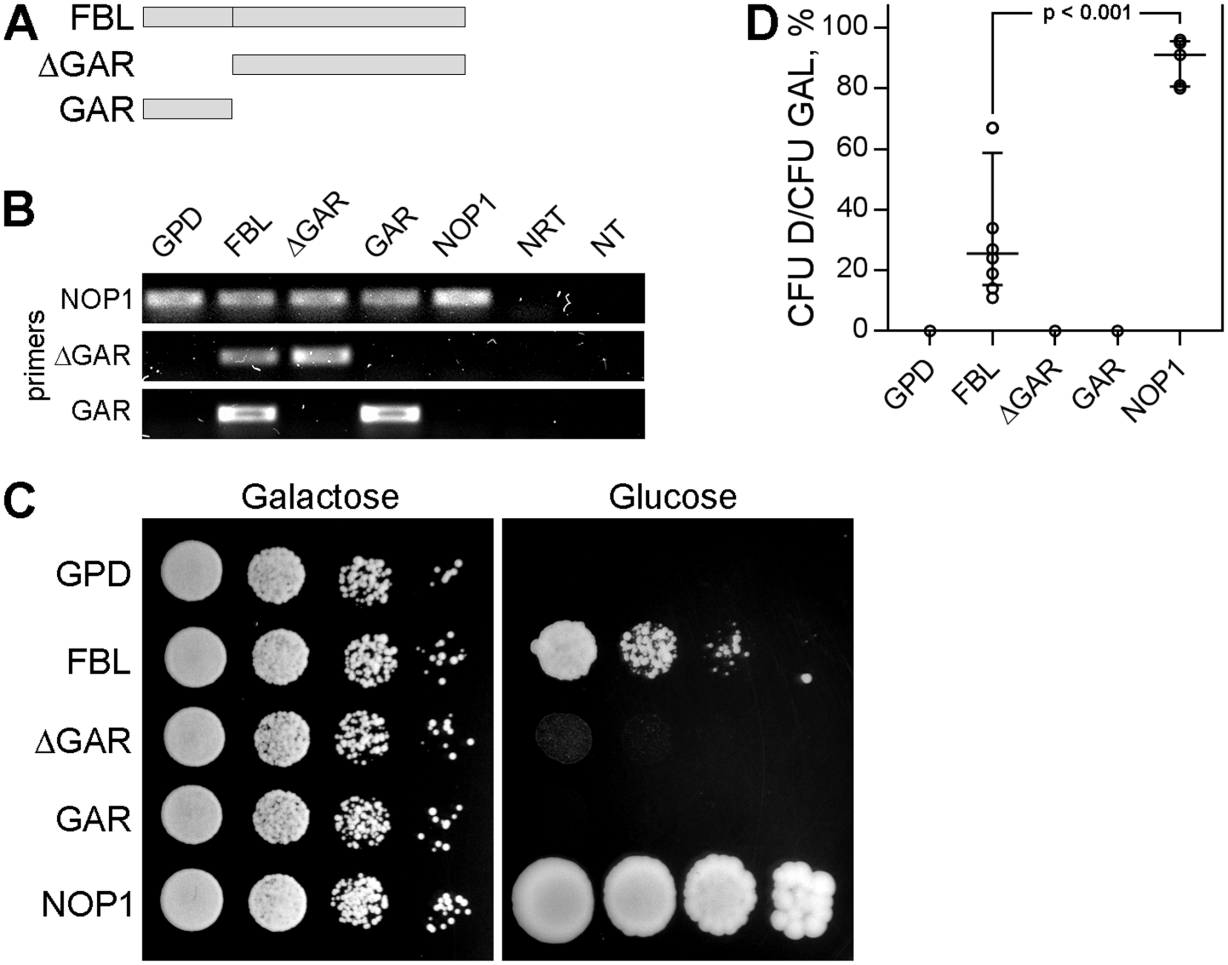
The GAR domain is necessary for FBL function. (A) A schematic representation of full-length fibrillarin (FBL) and its domains (ΔGAR, GAR). (B) Detection of NOP1, FBL, ΔGAR and GAR transcripts by RT-PCR analysis of different yeast strains coexpressing endogenous NOP1 and exogenous FBL, ΔGAR, GAR or NOP1. The primers against regions in yeast NOP1 human ΔGAR and GAR were designed and used. FBL transcripts were detected using primers against both GAR and ΔGAR. Two negative controls—no reverse transcriptase control (NRT) and no template control (NT), were added. (C) Yeast complementation assay. Coexpression of FBL, ΔGAR, GAR or NOP1 did not influence the survival of the GAL-NOP1 cells (Galactose). Expression of the full-length human FBL, but not ΔGAR or GAR, complemented NOP1 repression in *S. cerevisiae* (Glucose). For the negative control, GAL-NOP1 cells were transformed with the empty vector p416GPD. (D) Cell viability of the yeasts expressing FBL, ΔGAR, GAR, NOP1 or the empty vector p416-GPD after growing on glucose medium. Colony forming units (CFUs) were counted for each yeast strain. Yeast survival was estimated as the ratio of the cells on the glucose medium to the ratio of the cells on the galactose medium (CFU D/ CFU GAL). The comparison was performed using the Mann–Whitney test.

Expression of either human (Jansen et al., 1991), *Xenopus* (Jansen et al., 1991) or *Arabidopsis* (Barneche, Steinmetz & Echeverría, 2000) FBLs can complement yeast FBL, encoded by the *nop1* gene, indicating outstanding conservation of FBL function during evolution. Here, we used a yeast complementation assay to ascertain whether the GAR domain, which is specific for eukaryotic FBL, is necessary for FBL functioning. To regulate the expression of yeast NOP1, we put a *nop1* gene under the regulation of the galactose promoter. This GAL-NOP1 strain was transformed with plasmids encoding either human FBL or its truncated mutants—the GAR domain or FBL with the GAR domain deleted (ΔGAR) (Fig. 1A)—under the constitutively active glyceraldehyde-3-phosphate dehydrogenase (GPD) promoter. The plasmid encoding NOP1 under GPD promoter was used as a positive control.

While growing on galactose medium, cells simultaneously expressed both NOP1 and human FBL or its mutants (GAR or ΔGAR) (Fig. 1B), and the expression of the human protein did not affect the growth of the yeast strains (Fig. 1С, left panel). After transfer to glucose medium, the expression of endogenous yeast NOP1 was blocked, which was lethal for the GAL-NOP1 yeast cells (Fig. 1C, right panel). Expression of NOP1 from the GPD-NOP1 plasmid rescued nonviable yeast cells lacking the endogenous expression of NOP1. Full-length human FBL also complemented the mutant strain, but the percentage of the surviving cells was significantly lower than it was for the case of complementation with yeast NOP1 (Figs. 1C and 1D). Neither the GAR domain nor ΔGAR complemented NOP1, indicating that the GAR domain is necessary for the functional activity of FBL (Figs. 1C and 1D).

### The GAR domain is necessary for nuclear import of FBL

**Figure 2:**
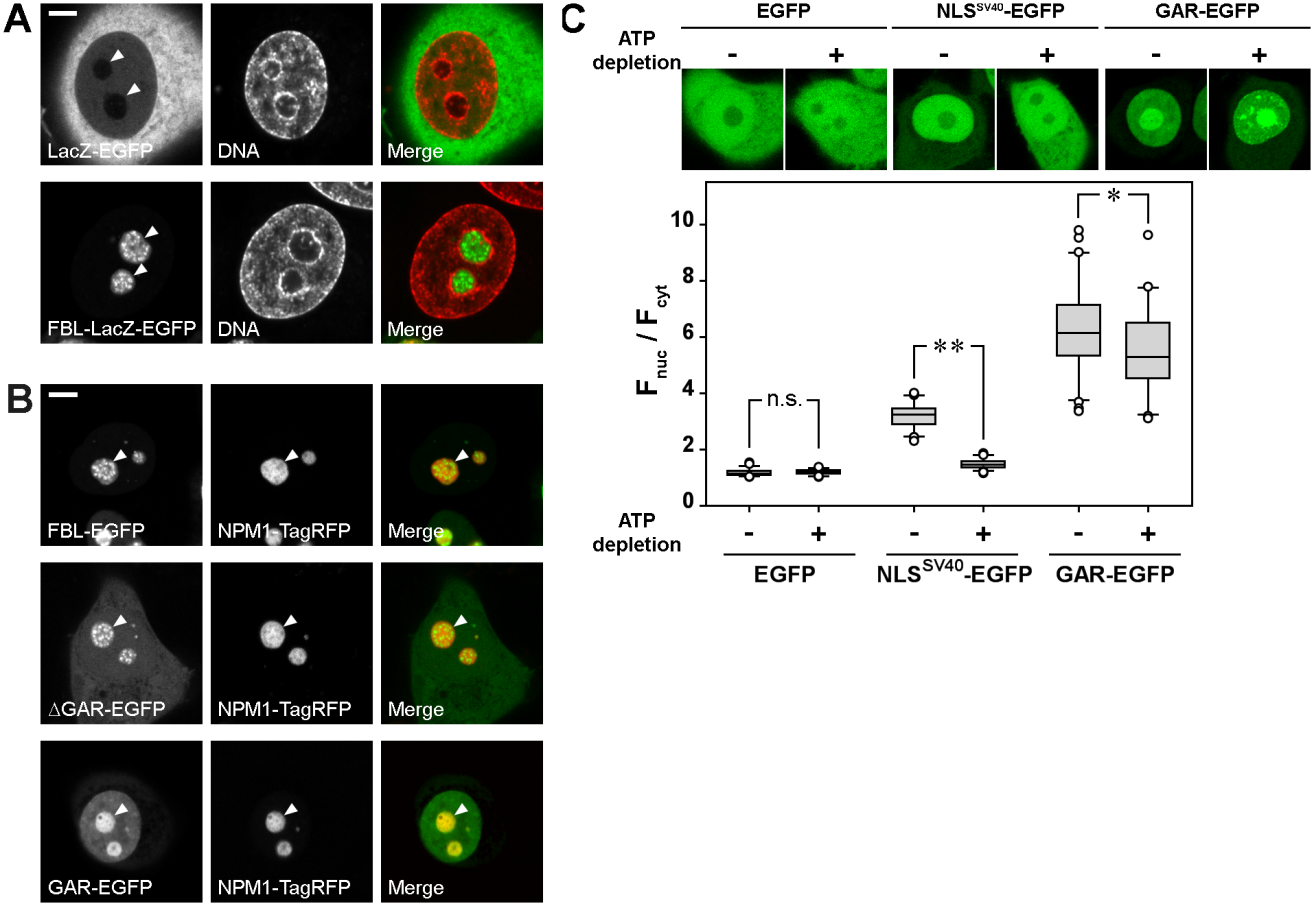
The GAR domain contains a nuclear localization signal (NLS). (A) FBL can traffic large cytoplasmic proteins (e.g., LacZ) into the nucleus of living HeLa cells. LacZ-EGFP fusion protein has an exclusively cytoplasmic localization (LacZ-EGFP, DNA, Merge), whereas the FBL-LacZ-EGFP fusion protein exhibits nucleolar localization similar to that of FBL-EGFP (FBL-LacZ-EGFP, DNA, Merge). Nucleoli are marked by arrowheads. DNA was stained with DAPI. (B) Localization of FBL-EGFP, ΔGAR-EGFP and GAR-EGFP in living HeLa cells (confocal microscopy). (C) ATP depletion decreased the accumulation of GAR-EGFP in the nucleus, revealing the energy-dependent nature of the nuclear import of this protein. EGFP was used as a negative control and NLS from the T antigen of the SV40 virus fuzed with EGFP (NLS^SV40^-EGFP) was used as a positive control. Box plots show the F_nuc_/F_cys_ ratios. Horizontal lines represent the median. n.s, not significant; *p < 0.05; **p < 0.001 (Mann–Whitney test, *n* > 50). Bars, 5 µm.

It was previously suggested that the GAR domain has NLS function (Snaar et al., 2000). Proteins with NLS capacity are able to transfer the large cytoplasmic proteins fuzed with them to the nuclei. Therefore, we constructed a plasmid encoding FBL fuzed with the β-galactosidase (*LacZ*) gene and EGFP (FBL-LacZ-EGFP with molecular weight of 177.25 kDa). The LacZ protein fuzed with EGFP predominantly localized to the cytoplasm (Fig. 2A, top panels), while the chimeric FBL-LacZ-EGFP fusion protein accumulated in the nuclei and was preferentially localized inside the nucleoli (Fig. 2A, bottom panels), indicating that FBL is capable of inducing the nuclear import.

To determine which FBL domain contains the NLS(s), we analyzed the localization of FBL-EGFP, ΔGAR-EGFP and GAR-EGFP in living HeLa cells (Fig. 2B). FBL-EGFP predominantly accumulated in the nucleoli. ΔGAR-EGFP also accumulated in nucleoli but was additionally distributed through the nucleoplasm and cytoplasm, indicating that the deletion of the GAR domain led to a defect in nuclear import. GAR-EGFP was predominantly localized in the GC of the nucleolus and nucleoplasm, and only weak fluorescence was detected in the cytoplasm. Thus, the GAR domain contributed to the accumulation of FBL in the nucleus.

FBL is a relatively small protein (<40 kDa), and therefore, it can potentially diffuse through nuclear pore complexes. In this situation, nuclear accumulation can be driven either by an active NLS-dependent import mechanism or by nuclear retention via interactions with intranuclear components. NLS-dependent nuclear import is an energy-dependent process (Richardson et al., 1988; Fried & Kutay, 2003), and ATP depletion leads to a rapid shutdown of NLS-mediated import (Shulga et al., 1996; Schwoebel, Ho & Moore, 2002). We depleted intracellular ATP and evaluated the nuclear accumulation of GAR-EGFP. As a negative control, EGFP was used; as a positive control, we used the NLS from the large T antigen of the SV40 virus fuzed with EGFP (NLS^SV40^-EGFP). To estimate nuclear accumulation, the ratio of nucleoplasmic fluorescence to cytoplasmic fluorescence (F_nuc_/F_cyt_) was measured. ATP depletion did not affect the localization of EGFP, while a twofold decrease in nuclear accumulation (F_nuc_/F_cyt_) was observed for NLS^SV40^-EGFP (Fig. 2C). A significant decrease in the nuclear accumulation of GAR-EGFP was detected after ATP depletion, but this decrease was not as robust as it was for NLS^SV40^-EGFP. Thus, the GAR domain has an NLS function by which FBL accumulates inside the nuclei; however, some accumulation is most likely due to nuclear retention.

### The GAR domain induces FBL accumulation in the GC of the nucleoli

**Figure 3:**
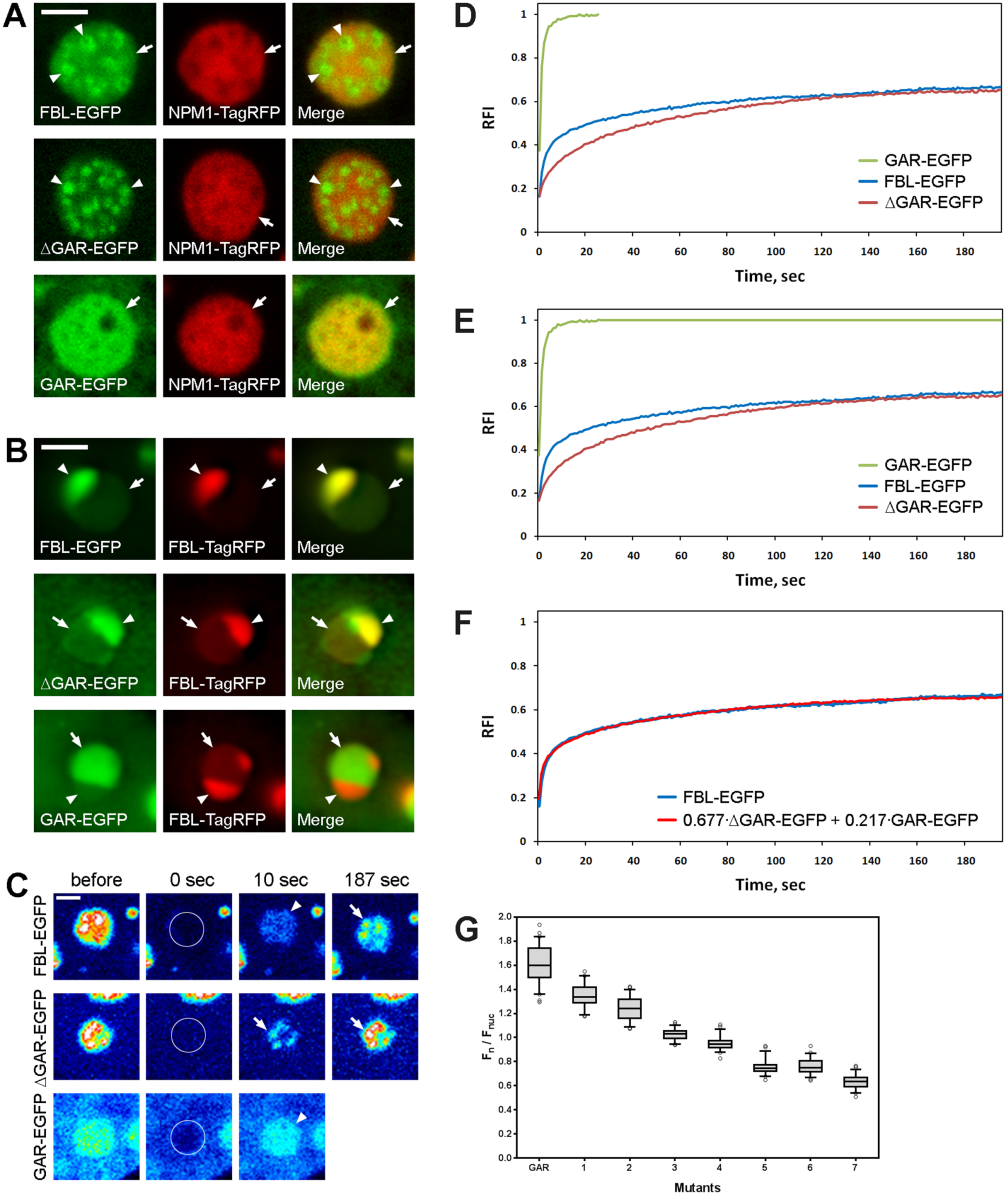
The GAR domain contains a nucleolar localization signal (NoLS). (A) Localization of FBL-EGFP, ΔGAR-EGFP, or GAR-EGFP and nucleolar protein NPM1-TagRFP in the nucleoli of living HeLa cells (fragments of interphase nuclei). NPM1-TagRFP was used as a nucleolus marker. General views of cells expressing FBL-EGFP, ΔGAR-EGFP or GAR-EGFP are presented in Fig. 2B. DFC is marked with arrowheads, GC is marked with arrows. (B) Localization of FBL-EGFP, ΔGAR-EGFP and GAR-EGFP in living HeLa cells after transcription was inhibited by ActD (0.1 µg/ml) for 4 h (fragments of interphase nuclei). These EGFP-fused proteins were coexpressed with FBL fuzed with TagRFP, which enabled the detection of changes in localization induced by the truncation. Residuals of the GC component are marked with arrows and residuals of the DFC are marked with arrowheads. (C) FRAP analysis of the FBL interactions with the DFC and GC in the nucleoli. Cells expressing FBL-EGFP, ΔGAR-EGFP or GAR-EGFP were imaged before and after photobleaching (0, 10 and 187 s). Fluorescence intensities are shown in pseudocolors using warm and cold colors for high and low signal intensities, respectively. (D) Recovery curves of FRAP experiments with FBL-EGFP, ΔGAR-EGFP and GAR-EGFP (HeLa cells). The FRAP curve represents an average of ~15 cells. (E) The curves of FBL-EGFP, ΔGAR-EGFP and GAR-EGFP (extrapolated curve), which were used for the construction of the FBL-EGFP curve from the ΔGAR-EGFP and GAR-EGFP curves. (F) The experimental and reconstructed FRAP curves of FBL-EGFP. (G) Nucleolar accumulation (F_n_/F_nuc_) of the GAR domain and the mutated forms of the GAR domain, in which different numbers of arginine residues were substituted with alanine residues (Fig. S1). Replacement of charged amino acids (R) with uncharged (A) decreased the F_n_/F_nuc_ in proportion to the number of substituted arginine residues. Nucleolar accumulation of all mutated GAR domains was statistically significantly different from that of non-mutated GAR domain (Mann–Whitney test, *p* < 0.001; *n* > 50). Bars, 2 µm.

Inside nucleoli, both FBL-EGFP and ΔGAR-EGFP were preferentially localized in small dot-like or ring-like intranucleolar structures, which most likely corresponded to DFC (Fig. 3A). It should be stressed that FBL was also clearly visible in the GC, where it was colocalized with co-expressed NPM1-TagRFP (Fig. 3A, top panels). ΔGAR-EGFP was also detected both in the DFC and in the GC, but the fluorescence in the GC was weaker than that of FBL-EGFP, indicating that ΔGAR-EGFP accumulation in the GC was substantially lower compared to that of FBL-EGFP (Fig. 3A, middle panels). GAR-EGFP was localized exclusively in the GC of the nucleoli (Fig. 3A, bottom panels). After segregation of the nucleolar components by actinomycin D (ActD), FBL-EGFP and ΔGAR-EGFP were found preferentially in the cap structure, i.e., in the DFC (Fig. 3B). In contrast, GAR-EGFP was colocalized with NPM1-TagRFP in the central part of the segregated nucleoli, i.e., in the residual GC (Fig. 3B).

It seems that the double localization of FBL (in the DFC and the GC) is driven by (i) the MT domain (ΔGAR), which is responsible for the accumulation inside the DFC and (ii) the GAR domain, which is responsible for the accumulation inside the GC. These two types of nucleolar accumulation can be driven by different types of interactions and therefore, we used FRAP for the investigation of FBL binding with the DFC and the GC in the nucleoli. After photobleaching, the fluorescence of FBL-EGFP was rapidly recovered in the GC of the nucleolus, and then it was slowly recovered in the DFC (Fig. 3C, top panels). Fluorescence recovery of ΔGAR-EGFP was detected exclusively in the DFC and was visible even at the short time after photobleaching (Fig. 3C, middle panels). It seems that similar recovery of FBL-EGFP in DFC may be masked by rapid recovery in GC. In the case of the GAR domain, rapid and complete recovery of fluorescence in the GC was observed (Fig. 3C, bottom panels).

Additionally, we analyzed the fluorescence recovery curves (Fig. S2). The half-time of fluorescence recovery (*t*_1/2_) of FBL-EGFP was ∼8 s (Fig. 3D). In the case of ΔGAR-EGFP, the *t*_1/2_ was substantially higher (~21 s), indicating a slower exchange of protein between the nucleolus and nucleoplasm compared to that of FBL-EGFP (Fig. 3D). In the case of GAR-EGFP, we observed an extremely fast fluorescence recovery in the GC of the nucleolus, with the *t*_1/2_ being ~0.9 s (Fig. 3D). We proposed that the fluorescence recovery curve of FBL-EGFP consists of two components with low and high dynamics—the former, slower recovery is caused by ΔGAR, and the second, faster recovery is caused by GAR. To test the hypothesis that two components contributed to the phenomenon represented by the recovery curve of FBL-EGFP, we approximated the FBL-EGFP curve as a weighted sum of the ΔGAR and the extrapolated GAR curves (Fig. 3E):

$$\left\{\widehat{FBL} \right\} = a \cdot \Delta {\rm GAR} + b \cdot {\rm GAR}$$

We used the Nelder–Mead method (Lagarias et al., 1998) to estimate the weights of *a* and *b*. The reconstructed FRAP $\left\{\widehat{FBL} \right\}$ curve was approximated as an initial FRAP FBL curve with RMSE = 0.051. The experimental and reconstructed FRAP FBL curves are shown in Fig. 3F.

Thus, it seems that FBL localization is relies two components and that the GAR domain is responsible for the highly dynamic accumulation of FBL in the GC of the nucleolus, while ΔGAR is responsible for the slower accumulation of the protein in the DFC.

Additionally, we analyzed the nucleolar accumulation of GAR domains based on the number of arginine residues that were substituted with alanine residues (R⇒A) and found a gradual decrease in the accumulation of the mutant GAR domains with an increase in the number of the substituted arginine residues (Fig. 3G). The more arginine residues that were replaced by uncharged alanine residues, the less efficient was the accumulation of the mutant GAR domains in the nucleolus.

### Methylation of arginine modulates the functions of the GAR domain

**Figure 4:**
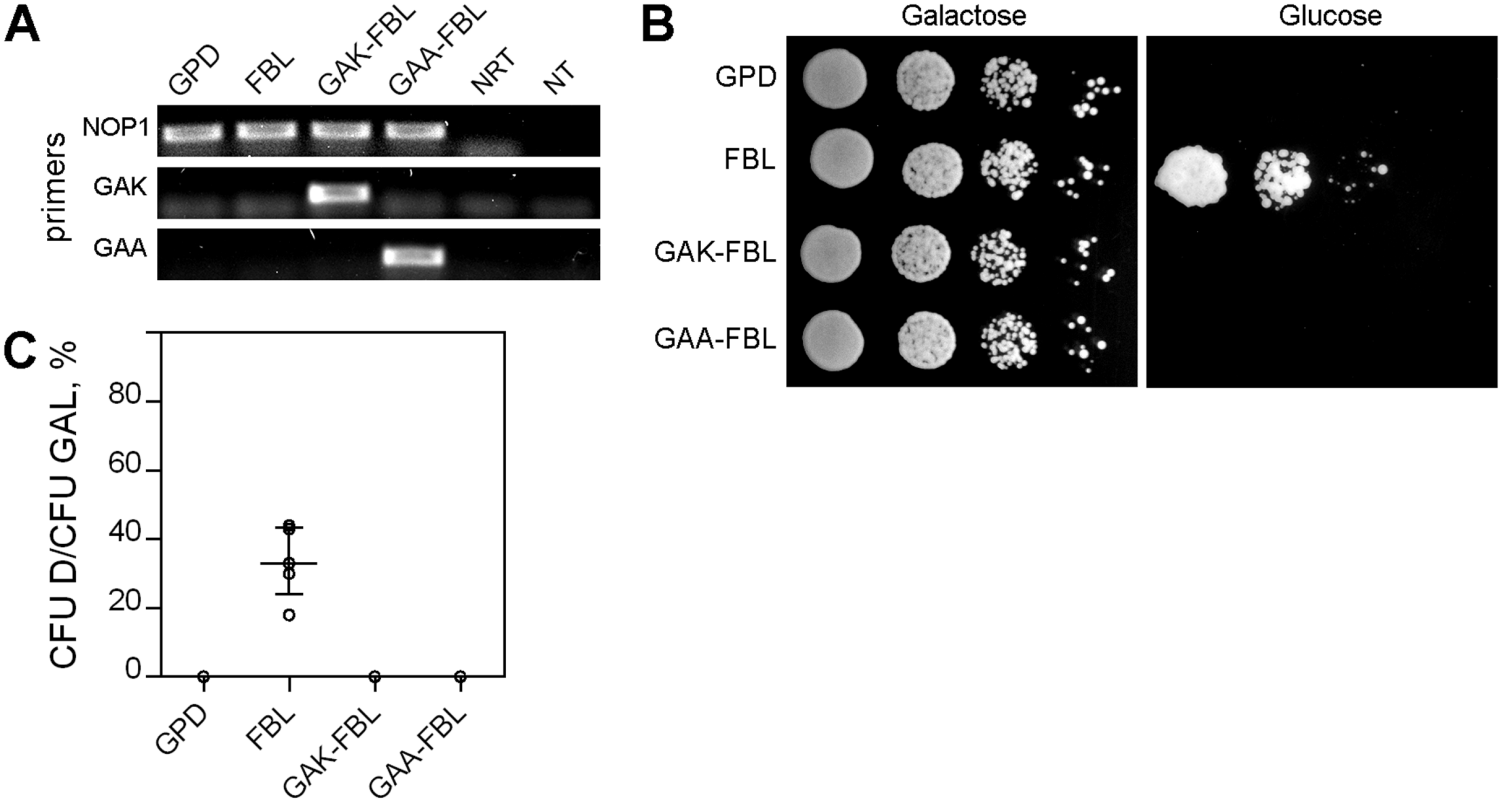
Arginine methylation influences FBL functioning. (A) Detection of NOP1, FBL, GAK-FBL and GAA-FBL transcripts by RT-PCR analysis in different yeast strains coexpressing endogenous NOP1 and exogenous FBL, GAK-FBL or GAA-FBL. The primers against regions in yeast NOP1 and the human GAR and mutated GAR domains (i.e., the GAK and GAA domains) were used. (B) Coexpression of the unmethylated forms of FBL (GAK-FBL and GAA-FBL) did not influence the survival of the GAL-NOP1 cells (Galactose). Expression of the full-length human FBL but not GAK-FBL or GAA-FBL complemented NOP1 repression in *S. cerevisiae* (Glucose). (C) Cell viability of the yeast cells expressing FBL, GAK-FBL, GAA-FBL, or the empty vector p416GPD after growth on glucose medium. Colony forming units (CFUs) were counted for each yeast strain. Yeast survival was estimated as the ratio of the cells on the glucose medium to the ratio of the cells on the galactose medium (CFU D/CFU GAL).

According to published data, arginine residues in the GAR domain undergo posttranslational asymmetric dimethylation (Lischwe et al., 1985; Liu & Dreyfuss, 1995; Ai et al., 1999; Yagoub et al., 2015). To identify the functional role of arginine methylation, the W303 pGAL-NOP1 strain was transformed with plasmids encoding mutated full-length FBL in which all the arginine (R) residues in the GAR domain were replaced with lysine (K) or alanine (A) (pGAK-FBL and pGAA-FBL, respectively) (Fig. S1). Simultaneous expression of endogenous NOP1 and mutated human FBLs (Fig. 4A) in yeast cultured on galactose medium did not influence yeast growth (Fig. 4B, left panel). In the absence of endogenous yeast NOP1 on the glucose medium, neither GAK-FBL nor GAA-FBL complemented the mutated yeast strain (Figs. 4B, right panel, 4C).

**Figure 5:**
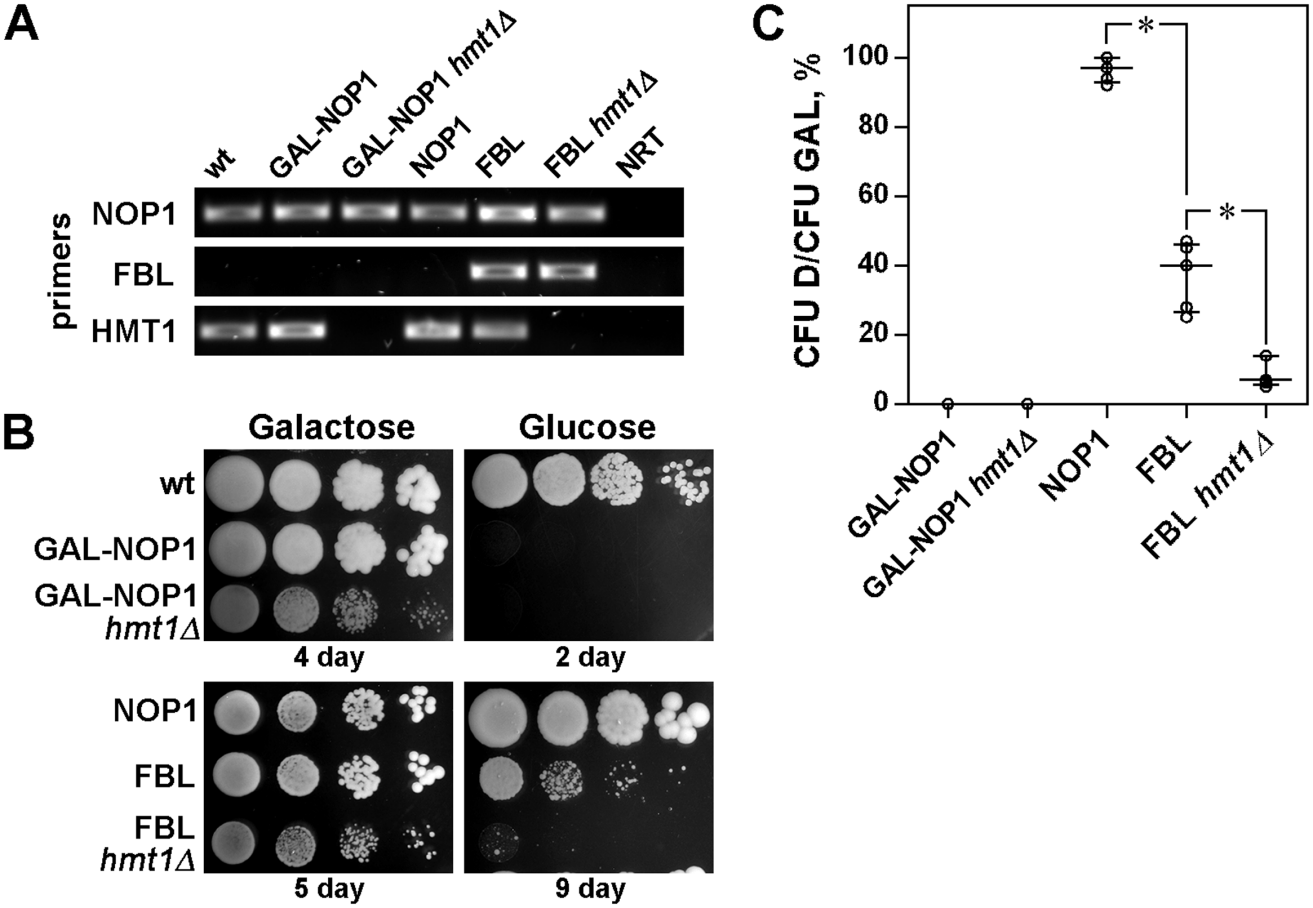
Arginine methylation by HMT1 influences FBL functioning. (A) Detection of NOP1, FBL and HMT1 transcripts by RT-PCR analysis in different yeast strains. The primers against regions in yeast NOP1, the human GAR and yeast HMT1 transcripts were used. (B) Coexpression of FBL did not influence the survival of the GAL-NOP1 cells, as well as deletion of *hmt1* gene (Galactose). Expression of the full-length human FBL complemented NOP1 repression in both GAL-NOP1 and GAL-NOP1 *hmt1*Δ straines (Glucose). (C) Cell viability of the yeast cells expressing NOP1 or FBL in either GAL-NOP1 or GAL-NOP1 *hmt1*Δ straines after growth on glucose medium. Colony forming units (CFUs) were counted for each yeast strain. Yeast survival was estimated as the ratio of the cells on the glucose medium to the ratio of the cells on the galactose medium (CFU D/CFU GAL).

In *Saccharomyces cerevisiae*, NOP1 is methylated by HMT1 (heterogeneous nuclear ribonucleoprotein MT) (Xu et al., 2003). Therefore, we additionally analyzed the complementation of yeast NOP1 by human full-length FBL in a *hmt1Δ* strain (Fig. 5A). It should be noted that deletion of *hmt1* substantially depressed yeast cell growth (Fig. 5B, left panels), therefore we analyzed growth of all yeast strains during 9 days, that is, much longer compared to experiments illustrated in Figs. 1 and 4. We observed that full-length FBL complemented the mutant GAL-NOP1 cells even after deletion of *hmt1* gene, but the percent of surviving cells was significantly lower compared to cells without deletion of *hmt1* gene (Figs. 5B, left panels and 5C). This observation indicated that methylation is indeed necessary for proper FBL functioning. The decrease was not so strong as in case of the cells with the expression of GAK-FBL (Figs. 4B and 4C), probably, due to incomplete demethylation of FBL. Indeed, it was demonstrated that a yeast strain disrupted for HMT1 retains 15% arginine MT activity (Gary et al., 1996).

Thus, arginine methylation in the GAR domain seems to be involved in FBL functioning.

### Arginine methylation within the GAR domain influences the nuclear import of FBL

To investigate the role of arginine methylation on the nuclear import of FBL, HeLa cells were treated with the methylation inhibitor AdOx, which inhibits all SAM-dependent enzymatic reactions, including protein arginine methylation (Cheng & Roberts, 2001).

**Figure 6:**
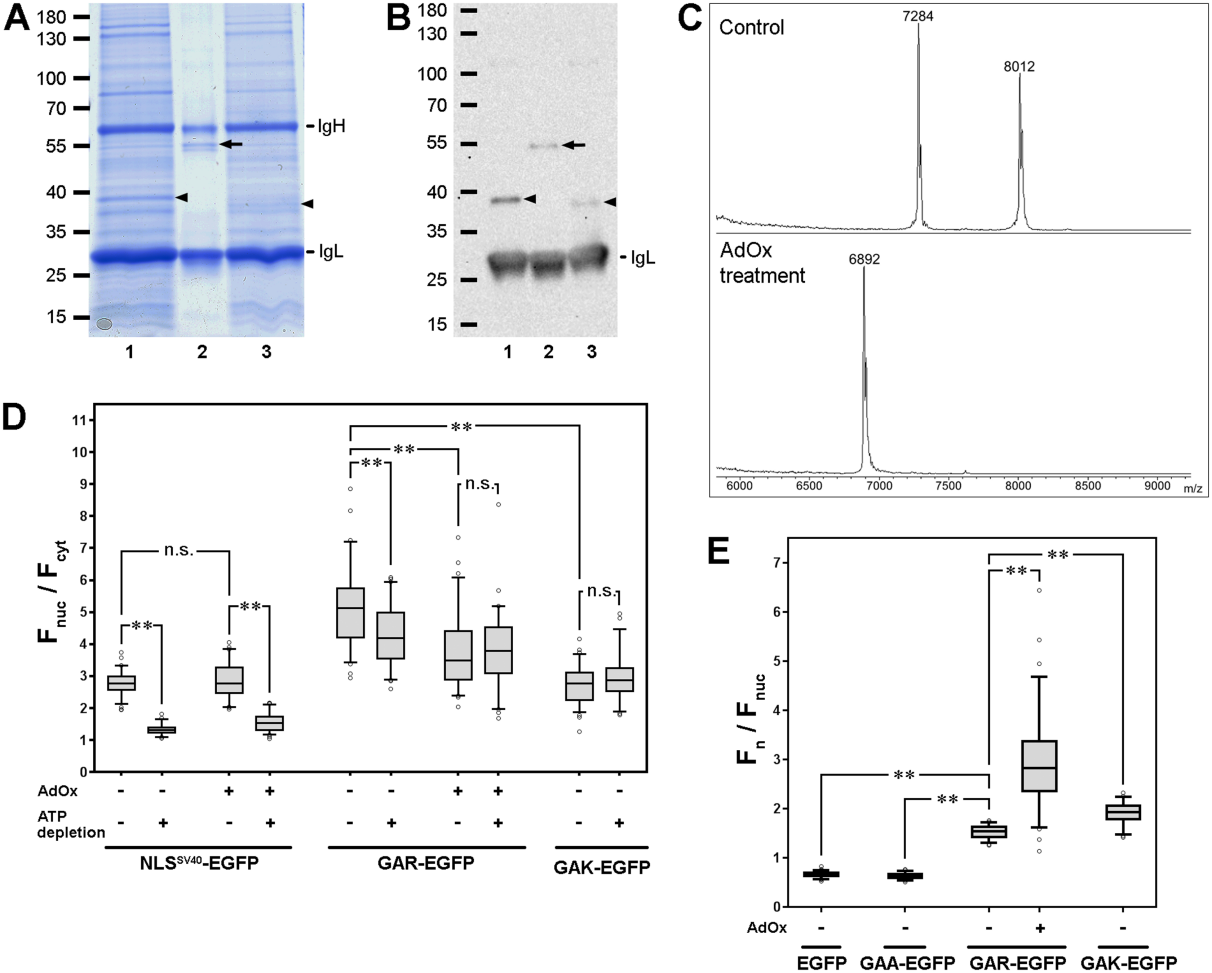
Arginine methylation influences FBL localization. (A) Immunoprecipitation of FBL-FLAG from untreated cells or cells treated with the methylation inhibitor AdOx (SDS-PAGE, Coomassie stain). The predicted FBL bands are marked with arrowheads. As a control, FLAG-BAP fusion protein (molecular weight 49.3 kDa) was immunoprecipitated. Line 1—HeLa cells expressing FBL-FLAG, line 2—FLAG-BAP fusion protein, line 3—HeLa cells expressing FBL-FLAG and treated with AdOx for 3 days before lysate preparation. (B) Immunoblotting for the detection of FBL-FLAG (anti-FLAG antibodies). (C) Fragmentation spectra of FBL digested with Lys-C in control cells (Control) or in cells treated with AdOx (AdOx treatment). (D) Arginine methylation influences the nuclear import of FBL. Treatment with AdOx did not influence nuclear localization (F_nuc_/F_cyt_) or the ability to react to ATP depletion of NLS^SV40^-EGFP. In contrast, AdOx treatment reduced the nuclear accumulation (F_nuc_/F_cyt_) of GAR-EGFP, and after this treatment, ATP depletion did not substantially influence the nuclear accumulation of GAR-EGFP, indicating that arginine methylation of the GAR domain is necessary for nuclear import. The substitution of arginine residues with lysine residues (R⇒K) also decreased nuclear accumulation. The GAK domain was insensitive to ATP depletion, similar to the GAR domain, in AdOx-treated cells. Box plots show the F_nuc_/F_cys_ ratios. Horizontal lines represent the median. The comparisons were performed using the Mann–Whitney test. n.s, not significant; **p < 0.001 (*n* > 50). (E) AdOx treatment increased the nucleolar (F_n_/F_nuc_) accumulation of GAR-EGFP and the unmethylated variant of the GAR domain (GAK domain) was accumulated to higher levels inside the nucleoli compared to the accumulation level of the methylated GAR domain, findings that indicate that arginine methylation impairs the NoLS function of the GAR domain. Box plots show the F_n_/F_nuc_ ratios. Horizontal lines represent the median. The comparisons were performed using the Mann–Whitney test. n.s, not significant; **p < 0.001 (*n* > 50).

To estimate the efficiency of the AdOx treatment, we used cells stably expressing FBL-FLAG. FBL-FLAG was immunoprecipitated from control cells and cells which were treated with AdOx. The bands containing FBL-FLAG were visible both in Coomassie-stained gels (Fig. 6A) and with immunoblot detection of FLAG (Fig. 6B). The band containing FBL-FLAG from AdOx-treated cells was slightly lower than that from untreated cells, possibly due to the absence of methyl groups after AdOx treatment. To confirm this suggestion, we analyzed samples using MALDI MS. We digested the selected bands with trypsin and identified the presence of FBL in these samples. Identification was reliable (score = 150). It is possible to use trypsin treated samples for identification of arginine methylation (Guo et al., 2014; Hart-Smith et al., 2012). In the N-terminal GAR domain of FBL there are 16 tryptic sites up to position 85, of which 15 are arginine residues and 1 lysine residue. If arginine residues are modified, the first tryptic peptide 1–77 should be of about 7 kDa. If arginine residues are not modified, a set of very short fragments should be obtained. We were able to identify long methylated peptide in control samples (Fig. S3) and only two short peptides in AdOx-treated samples (Fig. S4). But it seems that this analysis did not allowed us to estimate the efficiency of AdOx treatment. Therefore, we treated the samples with Lys-C proteinase, which cleaves the fragment of the GAR domain (1–77 a.a.) independent of the methylation status of the arginine residues. On the mass spectrum of AdOx-untreated control sample, we observed peak with average MH+ of 7,284 (Fig. 6C, top panel). The estimated m/z of the unmodified peptide 1–77 is 6,892, and thus, the shift associated with the modifications was 392 Da. Since 14 arginine residues are present in this peptide, all of them should be dimethylated as 392 = 14·28. The peak with MH+ of 8,012 was also present on the mass spectrum of this sample. It would correspond to the peptide 1–84 with one missed cleavage if the shift would be the same (predicted MH+ of 7,620 + 392). FBL region from 77 to 82 a.a. contains another one arginine residue, but it was not modified. After AdOx treatment, the only one peak with MH+ of 6,892, which corresponded to fully unmodified peptide 1–77 of FBL was detected (Fig. 6C, bottom panel). Thus, AdOx treatment effectively inhibited methylation of GAR-domain of FBL in HeLa cells.

AdOx treatment did not influence the nuclear accumulation of NLS^SV40^-EGFP (Fig. 6D), indicating that the inhibition of general protein methylation had no effect on the nuclear import system per se. In contrast, the nuclear accumulation of the GAR domain was decreased upon AdOx treatment (Fig. 6D). Importantly, after AdOx treatment, ATP depletion did not decrease the nuclear accumulation of GAR-EGFP, indicating that arginine methylation was necessary for active nuclear import but not for energy-independent nuclear retention. Additionally, to exclude the possibility that AdOx had an indirect effect on nuclear import, the localization of a genetically modified GAR domain, in which all the arginine residues were replaced with positively charged lysine residues (R⇒K), was analyzed (GAK domain). The nuclear accumulation (F_nuc_/F_cyt_) of GAK-EGFP was lower than that of GAR-EGFP in the AdOx-treated cells, and ATP depletion did not decrease the nuclear accumulation of GAK-EGFP (Fig. 6D). Thus, the methylation of arginines within the GAR domain was necessary for its accumulation in the nucleus via an active, energy-dependent mechanism but did not influence the accumulation mediated by nuclear retention.

### Arginine methylation negatively influences accumulation in the nucleolus

Upon treatment of the HeLa cells with AdOx, the efficiency of GAR-EGFP nucleolar accumulation (F_n_/F_nuc_) increased twofold compared to the efficiency of the untreated cells (Fig. 6E). Replacing all the arginine residues with lysine residues within the GAR domain also increased its accumulation in the nucleolus but less effectively than the AdOx treatment. Thus, the methylation of arginine seems to reduce the efficiency of the nucleolar accumulation of the GAR domain.

## Discussion

The MT domain of FBL is an example of an extremely conserved protein domain, in which the amino acid sequence was not substantially modified during evolution from *Archaea* to modern *Eukaryota* (see reviews: Rodriguez-Corona et al., 2015; Shubina, Musinova & Sheval, 2016). This exciting conservation of amino acid sequences leads to questions about possible problems caused by the evolution of the highly compartmentalized internal environments typical of modern *Eukaryota*. The major differences between prokaryotic and eukaryotic cells, which are the focus of the current study, are (i) the presence of a semipermeable barrier between the cytoplasm and the nucleus, which consists of a nuclear envelope with embedded nuclear pore complexes and (ii) the formation of nuclear bodies—membrane-free nuclear structures inside which intranuclear processes are compartmentalized. The data presented here indicate that the MT domain cannot effectively accumulate inside nuclei or correctly localize inside nucleoli. It is likely that the conservativity of the MT domain could not adapt FBL to the eukaryotic cell organization; therefore, an additional GAR domain evolved.

Here, we demonstrate that the GAR domain is necessary for FBL functioning and, additionally, integrates two activities necessary for proper localization inside eukaryotic cells. It should be noted that functional activity of GAR domain was investigated using yeast complementation assay. In some works this experimental approach was used to demonstrate the conservation of FBL function (Jansen et al., 1991; Barneche, Steinmetz & Echeverría, 2000). Here, we adapted this approach for the investigation of functional significance of GAR domain. But in general, it is impossible to extrapolate the conclusions from yeast to human cells. The nuclear import machinery is not so conserve as FBL, therefore, the nuclear accumulation was investigated using human cells.

Nuclear accumulation of small proteins (<40–60 kDa), which can diffuse through nuclear pore complexes, can be mediated by either active nuclear import or by interactions with nuclear components (nuclear retention). The presented data indicate that, in the case of FBL, both mechanisms are used. Indeed, if nuclear accumulation of NLS^SV40^-EGFP decreased to the level of EGFP after inhibition of active nuclear import (ATP depletion), the accumulation of GAR-EGFP was slightly decreased, indicating that only some of the nuclear FBL accumulation relied on energy-dependent nuclear import. The important feature of this energy-dependent accumulation is that the methylation of arginine residues in the GAR domain was necessary.

The additional interaction of GAR with nuclear structures (e.g., with the GC of the nucleoli) can lead to energy-independent nuclear retention. The results from the FRAP experiments demonstrate that the retention of the GC was mediated by the extremely dynamic interactions of the GAR domain with GC components. The mechanism of this retention may be similar to those of NoLS-mediated nuclear accumulation. It has been demonstrated that protein accumulation in the GC of the nucleoli can be driven by protein regions enriched with positively charged amino acids (NoLS(s)), which electrostatically interact with nucleolar components (Musinova et al., 2011, 2015; Savada & Bonham-Smith, 2013; Martin et al., 2015). The accumulation of the GAR domain was decreased when charged amino acids were substituted by uncharged alanine residues (R⇒A); moreover, the decrease in accumulation was dependent on the number of replaced amino acids, indicating the likelihood that accumulation in the GC can also be driven by a charge-dependent mechanism.

Experiments in which protein methylation was inhibited demonstrated that the nucleolar accumulation mediated by the GAR domain seems to be suppressed by arginine methylation. Methylation does not change the charge of the sequence but increases the size of the modified residues and their hydrophobicity (Pahlich, Zakaryan & Gehring, 2006; Bedford & Clarke, 2009), which may lead to a decrease in nucleolar accumulation. Therefore, the accumulation of the GAR domain in which all the arginine residues are substituted by lysine residues (R⇒K) was substantially higher than that of the unmutated GAR domain. These data are in agreement with the observation that methylation can modulate nucleolar accumulation of some proteins (Tapia et al., 2010; Fulcher et al., 2016; Paris et al., 2018), including NOP1 in *S. cerevisiae* (Smith et al., 2020).

FBL functions preferentially in the DFC, but the accumulation in the GC via the GAR domain may be biologically significant. It is likely that FBL is involved in later stages of pre-rRNA processing. However, even if this accumulation was not accompanied by any biochemical reactions, the interactions with the components of the GC can lead to accumulation near the functional site (i.e., in the DFC). If this supposition is correct, then the additional FBL domain contributes to effective functioning not only by accumulating inside nuclei but also by accumulating in the GC of the nucleoli. These activities of the GAR domain may lead to the effective accumulation of dynamic proteins in the limited area of the DFC.

## Conclusions

**Figure 7:**
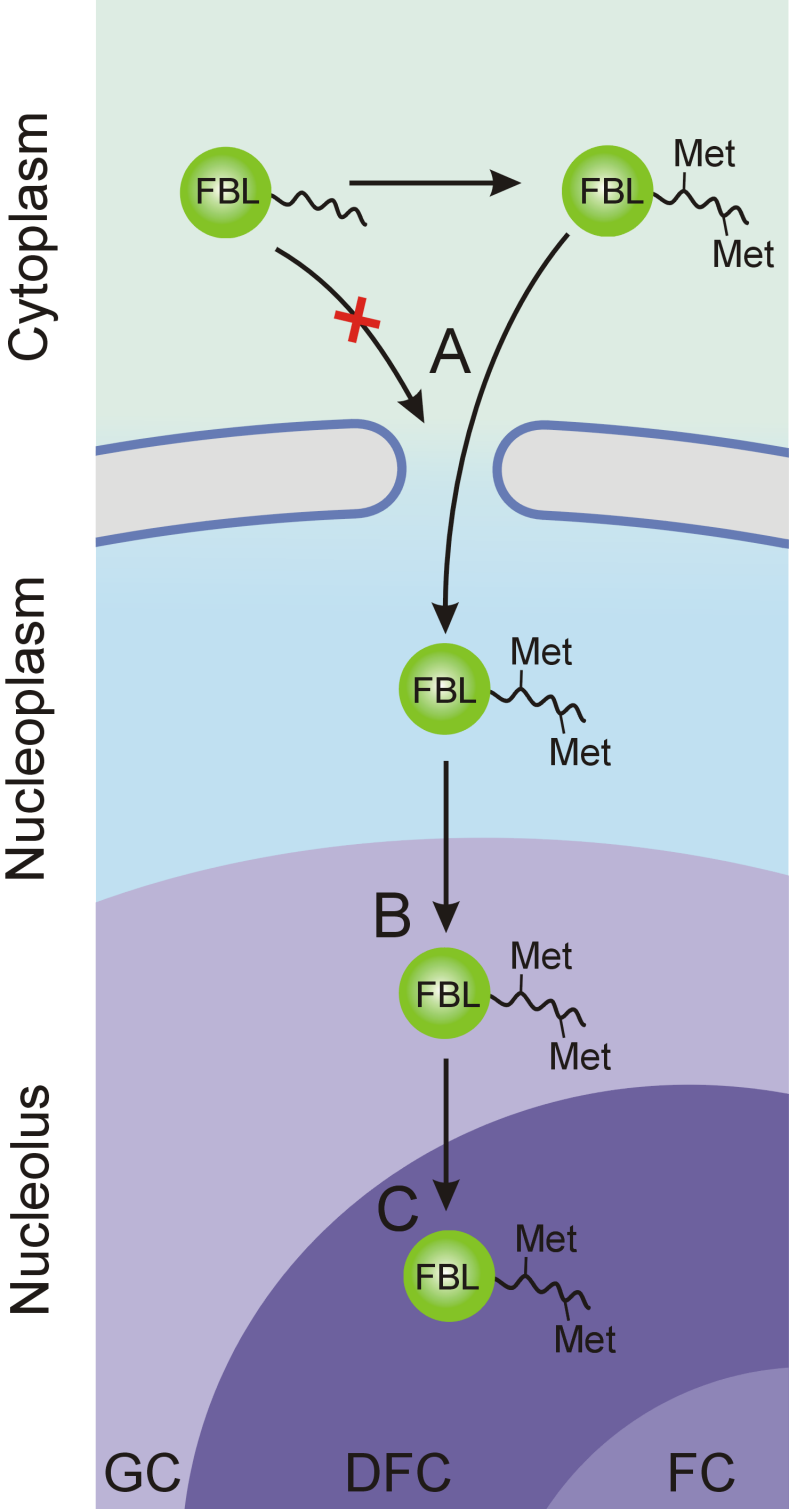
Stages of FBL transfer from the cytoplasm to intranucleolar transcription sites (in the DFC). (A) Methylation of the arginine residues of the GAR domain facilitates nuclear import of FBL molecules. (B) Dynamic accumulation of FBL mediated by the GAR domain led to the accumulation in the GC in the nucleoli, which likely increased the access to the intranucleolar transcription sites in the DFC. (C) Localization of FBL in the intranucleolar transcription sites in the DFC.

Thus, during the transition from *Archaea* to *Eukaryota*, FBL-like proteins acquired additional domain that functions as NLSs and NoLSs, leading to the evolution of the eukaryotic FBL. The possible principle of integration of different activities is summarized in Fig. 7. Several stages can be described based on the data obtained in the current study. (A) Arginine residues in the GAR domain of functionally active FBL are methylated. FBL is methylated preferentially by PRMT1 (Tang et al., 1998). According to published data, PRMT1 located partially in cytoplasm (Herrmann et al., 2005; Goulet et al., 2007; Herrmann & Fackelmayer, 2009), and, thus, can methylate FBL before import to the nuclei. Methylation is necessary for nuclear import (the NLS function of the GAR domain); therefore, one can assume that only functionally active FBL molecules with methylated arginine can be effectively trafficked to the nucleus. (B) FBL accumulates in the GC of nucleoli, probably via a charge-dependent mechanism, that is, functions as an NoLS. It is likely that FBL is involved in some stages of pre-rRNA processing, which occurs in the DFC region. However, even if this is not the case, the accumulation in the GC can enhance interactions with pre-rRNA transcripts on the border of the DFC and the FCs. Importantly, methylation decreased the NoLS activity of the GAR domain, and one can assume that unmethylated FBL is retained more strongly than necessary. (C) Finally, the GAR domain is necessary for FBL functioning. At this stage, methylation of arginine in the GAR domain seems to be essential for the accumulation in the DFC and, simultaneously, for functioning.

Thus, the GAR domain effectively adapted FBL to function inside highly compartmentalized eukaryotic cells and methylation of the GAR domain enables the optimization and integration of the different activities of this protein.

## Supplemental Information

10.7717/peerj.9029/supp-1 Supplemental Information 1 Primers used in this study.

10.7717/peerj.9029/supp-2 Supplemental Information 2 Figures S1–S4.

10.7717/peerj.9029/supp-3 Supplemental Information 3 Raw data for Fig. 1B.

10.7717/peerj.9029/supp-4 Supplemental Information 4 Raw data for Fig. 1C.

10.7717/peerj.9029/supp-5 Supplemental Information 5 Raw data for Fig. 4A.

10.7717/peerj.9029/supp-6 Supplemental Information 6 Raw data for Fig. 4B.

10.7717/peerj.9029/supp-7 Supplemental Information 7 Raw data for Fig. 5A.

10.7717/peerj.9029/supp-8 Supplemental Information 8 Raw data for Fig. 5B.

10.7717/peerj.9029/supp-9 Supplemental Information 9 Raw data for Fig. 6A.

10.7717/peerj.9029/supp-10 Supplemental Information 10 Raw data for Fig. 6B.

10.7717/peerj.9029/supp-11 Supplemental Information 11 Fig. 1D raw data. Cell viability of the yeasts expressing GPD-FBL, GPD-ΔGAR, GPD-GAR, GPD-NOP1 or the empty vector p416-GPD after growing on YPD plates. Colony forming units (CFUs) were counted for each yeast strain. Yeast survival was estimated as the ratio of the cells on the glucose medium to the ratio of the cells on the galactose medium (CFU D/CFU GAL). The comparison was performed using the Mann–Whitney test.

10.7717/peerj.9029/supp-12 Supplemental Information 12 Fig. 2C raw data. ATP depletion decreased the accumulation of GAR-EGFP in the nucleus, revealing the energy-dependent nature of the nuclear import of this protein. EGFP was used as a negative control, and NLS from the T antigen of the SV40 virus fuzed with EGFP (NLS^SV40^-EGFP) was used as a positive control. Box plots show the F_nuc_/F_cys_ ratios. Horizontal lines represent the median. n.s, not significant; *p < 0.05; **p < 0.001 (Mann–Whitney test, *n* > 50). Bars, 5 µm.

10.7717/peerj.9029/supp-13 Supplemental Information 13 Figures 3D, E, F raw data for ΔGAR curves. Recovery curves of FRAP experiments with FBL-EGFP, ΔGAR-EGFP and GAR-EGFP (HeLa cells). The FRAP curve represents an average of ~15 cells.

10.7717/peerj.9029/supp-14 Supplemental Information 14 Figures 3D, E, F raw data for GAR curves. Recovery curves of FRAP experiments with FBL-EGFP, ΔGAR-EGFP, and GAR-EGFP (HeLa cells). The FRAP curve represents an average of ~15 cells.

10.7717/peerj.9029/supp-15 Supplemental Information 15 Figures 3D, E, F raw data for FBL curves. Recovery curves of FRAP experiments with FBL-EGFP, ΔGAR-EGFP and GAR-EGFP (HeLa cells). The FRAP curve represents an average of ~15 cells.

10.7717/peerj.9029/supp-16 Supplemental Information 16 Fig. 3G raw data. Nucleolar accumulation (F_n_/F_nuc_) of the GAR domain and the mutated forms of the GAR domain, in which different numbers of arginine residues were substituted with alanine residues.

10.7717/peerj.9029/supp-17 Supplemental Information 17 Fig. 4C raw data. Cell viability of the yeast cells expressing GPD-FBL, GPD-GAK-FBL, GPD-GAA-FBL or the empty vector p416GPD after growth on YPD plates. Colony forming units (CFUs) were counted for each yeast strain. Yeast survival was estimated as the ratio of the cells on the glucose medium to the ratio of the cells on the galactose medium (CFU D/CFU GAL).

10.7717/peerj.9029/supp-18 Supplemental Information 18 Fig. 5C raw yeast. Cell viability of the yeast cells expressing GPD-NOP1, GPD-FBL or GPD-FBL in dHMT1 strain after growth on YPD plates. Colony forming units (CFUs) were counted for each yeast strain. Yeast survival was estimated as the ratio of the cells on the glucose medium to the ratio of the cells on the galactose medium (CFU D/CFU GAL).

10.7717/peerj.9029/supp-19 Supplemental Information 19 Fig. 6D raw data. Arginine methylation influences the nuclear import of FBL. Treatment with AdOx did not influence nuclear localization (F_nuc_/F_cyt_) or the ability to react to ATP depletion of NLS^SV40^-EGFP. In contrast, AdOx treatment reduced the nuclear accumulation (F_nuc_/F_cyt_) of GAR-EGFP, and after this treatment, ATP depletion did not substantially influence the nuclear accumulation of GAR-EGFP, indicating that arginine methylation of the GAR domain is necessary for nuclear import. The substitution of arginine residues with lysine residues (R⇒K) also decreased nuclear accumulation. The GAK domain was insensitive to ATP depletion, similar to the GAR domain, in AdOx-treated cells. Box plots show the F_nuc_/F_cys_ ratios. Horizontal lines represent the median. The comparisons were performed using the Mann–Whitney test. n.s. – not significant; **p < 0.001 (*n* > 50).

10.7717/peerj.9029/supp-20 Supplemental Information 20 Fig. 6E raw data. AdOx treatment increased the nucleolar (F_n_/F_nuc_) accumulation of GAR-EGFP, and the unmethylated variant of the GAR domain (GAK domain) was accumulated to higher levels inside the nucleoli compared to the accumulation level of the methylated GAR domain, findings that indicate that arginine methylation impairs the NoLS function of the GAR domain. Box plots show the F_n_/F_nuc_ ratios. Horizontal lines represent the median. The comparisons were performed using the Mann–Whitney test. n.s. – not significant; **p < 0. 001 (*n* > 50).

## Additional Information and Declarations

### Data Availability

The following information was supplied regarding data availability: The raw data are available in the Supplemental Files.
