# Supplementary material for: The GAR domain integrates functions that are necessary for the proper localization of fibrillarin (FBL) inside eukaryotic cells
Source: PeerJ. 2020 Apr 28;8:e9029. doi: 10.7717/peerj.9029 (PMC7194090; doi:10.7717/peerj.9029)
Supplement: Supplemental Information 1 [file peerj-08-9029-s001.docx]

**Supplementary Table S1. Primers used in this study.**

| **Plasmid** | **Sequence (5'-3')** |
| --- | --- |
| **Plasmids for expression in human cells** | |
| FBL-EGFP | AGTCGGATCCATGAAGCCAGGATTCAGTCCCC  AGTCAAGCTTTCAGTTCTTCACCTTGGGGGG |
| GAR-EGFP | AGTCGGATCCATGAAGCCAGGATTCAGTCCCC  AGTCAAGCTTTCAGTTTCCTCTTTTTCCTCCCCG |
| ΔGAR-EGFP | AGTCGGATCCATGCAGTCGGGGAAGAATGTGATGG  AGTCAAGCTTTCAGTTCTTCACCTTGGGGGG |
| pFBL-LacZ-EGFP | Amplification of *fbl*  AGTCAGATCTATGAAGCCAGGATTCAGTCCCCGTG  AGTCGTCGACTCGTTCTTCACCTTGGGGGGTGGCC  Amplification of *LacZ*  AGTCGGTACCTGCCGTCGTTTTACAACGTCG  AGTCGGATCCTCTTTTTGACACCAGACCAACTG |
| pLacZ-EGFP | ATAAAAGCTTATGTCGTTTACTTTGACCAACAA  ATAAGGATCCAATTTTTGACACCAGACCAACTG |
| pNLS^SV40-^EGFP | TCGACCATGTATCCTAAAAAAAAACGTAAAGTTGAAGATCCT  GATCAGGATCTTCAACTTTACGTTTTTTTTTAGGATACATGG |
| **Yeasts complementation test** | |
| pGAL-NOP1 | TACTACTGTTATATAAGTTATTCTTCGAGAAACAATTAGATATCATTCATCGGATGAATTCGAGCTCGTTTAAAC  AGCCACCTCTGGAACCTCCACGGGAACCACCTCTGCTACCTGGTCTGAATGACATTTTGAGATCCGGGTTTT |
| GPD-FBL | AGTCGGATCCATGAAGCCAGGATTCAGTCCCC  AGTCAAGCTTTCAGTTCTTCACCTTGGGGGG |
| GPD-GAR | AGTCGGATCCATGAAGCCAGGATTCAGTCCCC  AGTCAAGCTTTCAGTTTCCTCTTTTTCCTCCCCGAC |
| GPD-ΔGAR | AGTCGGATCCATGCAGTCGGGGAAGAATGTGATGG  AGTCAAGCTTTCAGTTCTTCACCTTGGGGGG |
| GPD-GAA-FBL | AGTCGGATCCATGAAGCCAGGATTCAGTCCCC  AGTCAAGCTTTCAGTTCTTCACCTTGGGGGGTGG |
| GPD-GAK-FBL | AGTCGGATCCATGAAGCCAGGATTCAGTCCCC  AGTCAAGCTTTCAGTTCTTCACCTTGGGGGGTGG |
| GPD-NOP1 | AGTCGGATCCATGTCATTCAGACCAGGTAG  AGTCAAGCTTTTATTTCTTCAAACCGCTTCT |
| HMT1-pUG | CACCTTGCCGTTTCCAAAAAAGAGTTAGAACCGACAAATTCATCCAAAGAAAATACAGCTGAAGCTTCGTACGC  AAGTTTGTTTATTTGCTTTTCAAATTTTTTTCTTTCTCCAGCAAACAAAAGTCGCATAGGCCACTAGTGGATCTG |
| **Primers for verifying of yeast strains by PCR** | |
| GAL-NOP1 | GTTTAAACGAGCTCGAATTC  TGTACTTGCTGATATTGTGG |
| *hmt1Δ* | GCGTACGAAGCTTCAGCTG  CTGAAGACATCCCATGTCCA |
| **Primers for RT-PCR** | |
| GAR | AAGCCAGGATTCAGTCCCC  CTCTTTTTCCTCCCCGACCA |
| ΔGAR | ACATCAAACCGGGGGCTAAG  ATTACGCAGGAAGGTGTGGG |
| NOP1 | GGCCCCAGGTGAATCAGTTT  CGGCGTAGACAACACCTTCT |
| GAA | GCCAGGATTCAGTCCCGC  TCCTCTTTTTCCTCCCGCAC |
| GAK | GCTTTGGTGACAAAGGTGGT  CTTTGTTGCCACCAGAATGG |
| HMT1 | TGAGCAAGACAGCCGTGAAA  CGATAACATGCTTCGCACCG |
