# Supplementary material for: The GAR domain integrates functions that are necessary for the proper localization of fibrillarin (FBL) inside eukaryotic cells
Source: PeerJ. 2020 Apr 28;8:e9029. doi: 10.7717/peerj.9029 (PMC7194090; doi:10.7717/peerj.9029)
Supplement: Supplemental Information 2 [file peerj-08-9029-s002.pdf]

Supplementary Figures

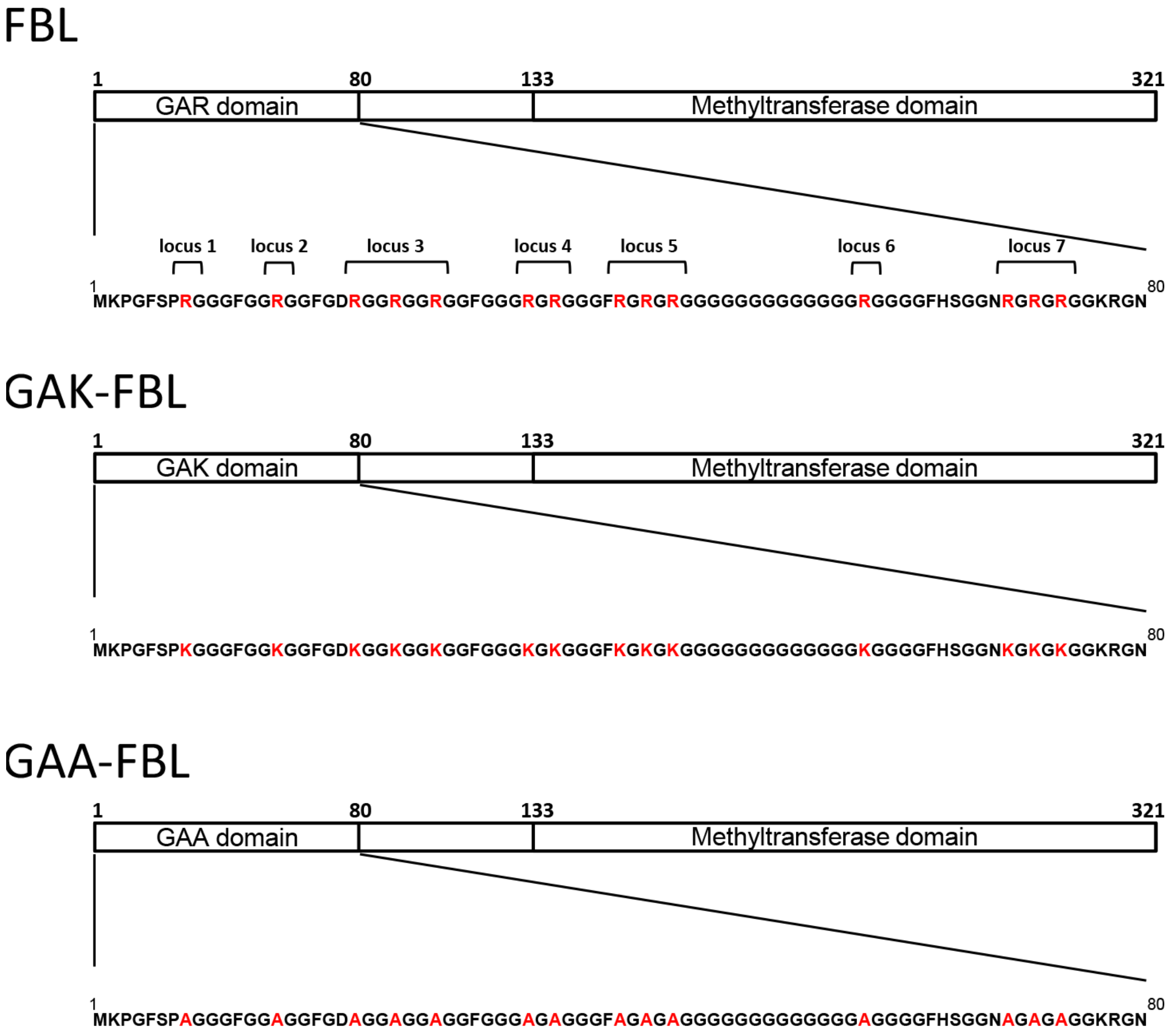

Supplementary Fig. S1. A schematic representation of full-length fibrillarin (FBL) and its GAR domain. The full-length FBL and two mutants are presented (GAK-FBL and GAA-FBL). The arginines in GAR domain of FBL, lysins in GAK domain of GAK-FBL and alanines in GAA domain of GAA-FBL are highlighted in red. The groups of arginines which were subsequently mutated in GAR domain of FBL are designated as locus 1, locus 2, etc.

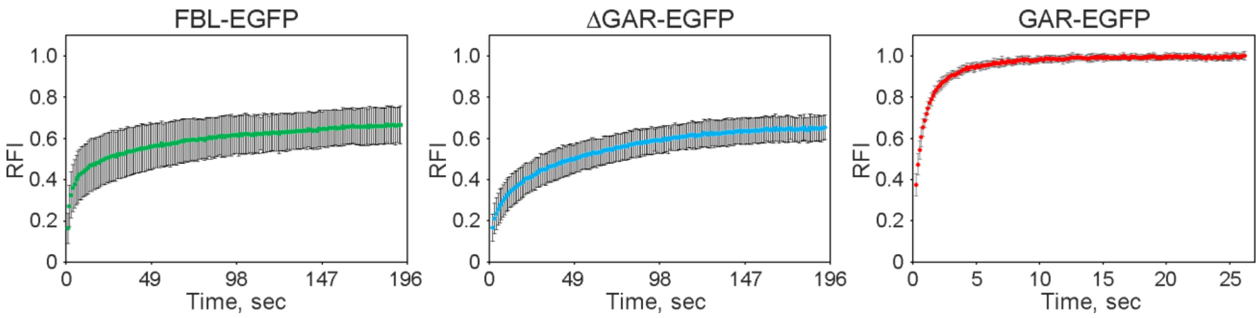

Supplementary Fig. S1. FRAP analysis of the FBL interactions with the DFC and GC in the nucleoli of HeLa cells. Recovery curves of FRAP experiments with FBL-EGFP, ΔGAR-EGFP, and GAR-EGFP. Each FRAP curve represents an average of ~15 cells (mean ± standard deviation).

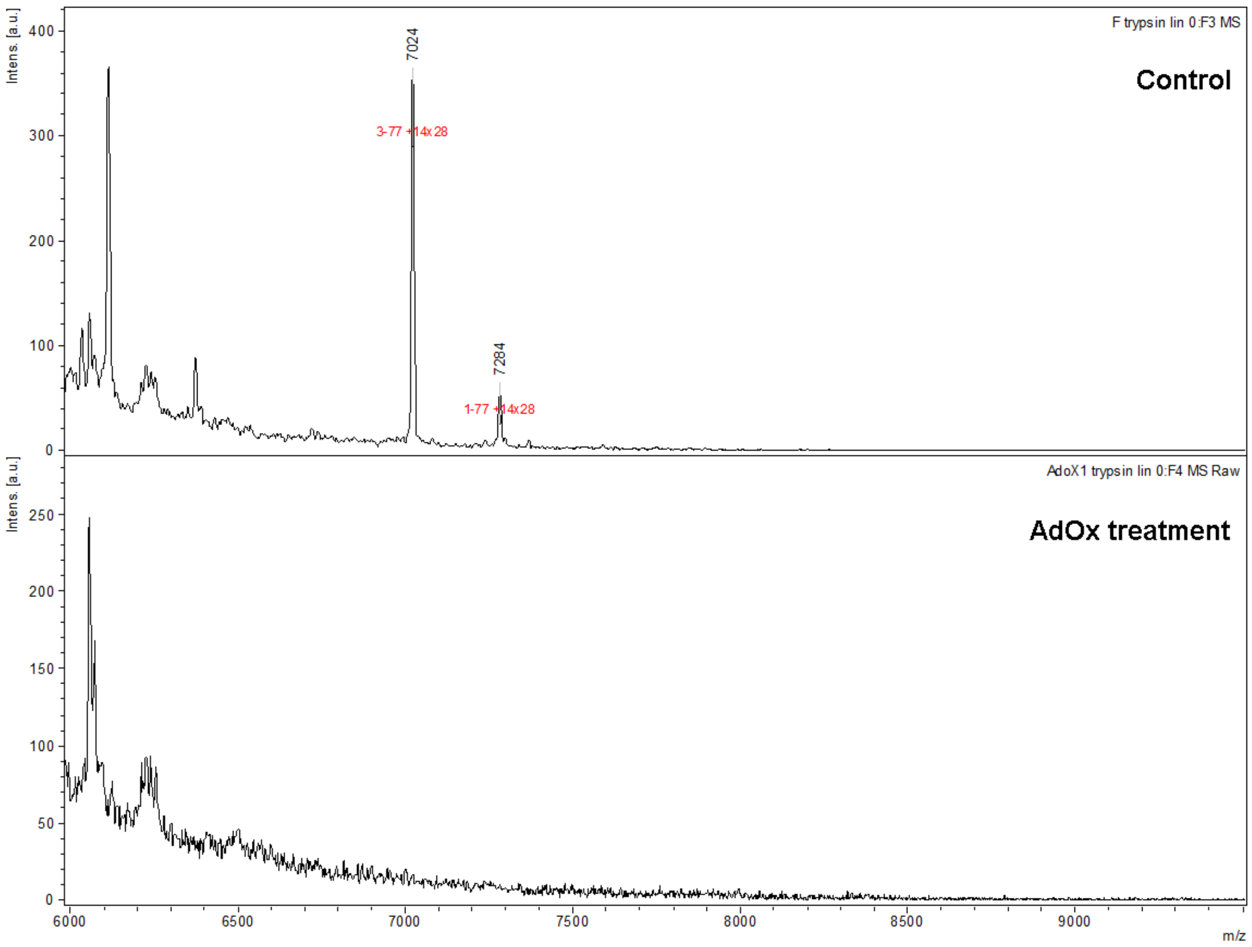

Supplementary Fig. S3. AdOx inhibits methylation of GAR domain of FBL in HeLa cells. Fragmentation spectra of FBL digested with trypsin. In linear regime, we observed a long peptide corresponding to the GAR domain in lysates of untreated cells (top panel), which corresponded to 1-77 a.a. of FBL (+14 dimethyls) (MW+ 7284). Additionally, the 3-77 peptide (+14 dimethyls) was detected (MW+ 7024). After AdOx treatment, these peptides (MW+ 7024 and 7284) were absent, indicating a loss of arginine methylation.

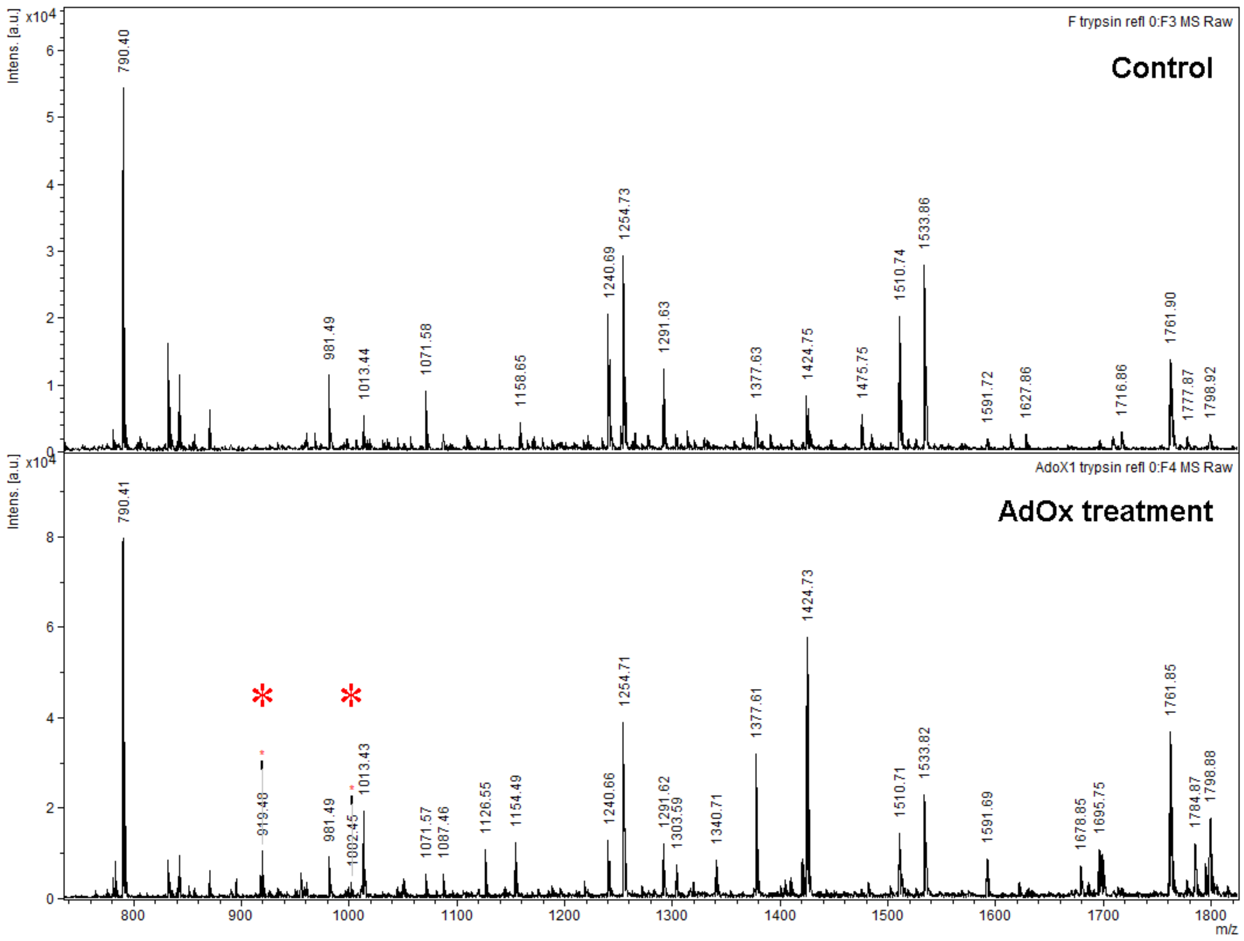

Supplementary Fig. S4. AdOx inhibits methylation of GAR domain of FBL in HeLa cells. Fragmentation spectra of FBL digested with trypsin in control cells (top panel) or in cells treated with AdOx (bottom panel). We were able to identify only two short peptides in AdOx-treated samples (red stars).
